# Metabolic rewiring during bone development underlies tRNA m^7^G–associated primordial dwarfism

**DOI:** 10.1172/JCI177220

**Published:** 2024-09-10

**Authors:** Qiwen Li, Shuang Jiang, Kexin Lei, Hui Han, Yaqian Chen, Weimin Lin, Qiuchan Xiong, Xingying Qi, Xinyan Gan, Rui Sheng, Yuan Wang, Yarong Zhang, Jieyi Ma, Tao Li, Shuibin Lin, Chenchen Zhou, Demeng Chen, Quan Yuan

**Affiliations:** 1 State Key Laboratory of Oral Diseases and National Center for Stomatology and National Clinical Research Center for Oral Diseases, West China Hospital of Stomatology, Sichuan University, Chengdu, China.; 2 Center for Translational Medicine, Precision Medicine Institute, The First Affiliated Hospital, Sun Yat-sen University, Guangzhou, China.; 3 West China–Washington Mitochondria and Metabolism Center and Department of Anesthesiology, West China Hospital, Sichuan University, Chengdu, China.; 4 Otorhinolaryngology Hospital, The First Affiliated Hospital, Sun Yat-sen University, Guangzhou, China.

**Keywords:** Bone biology, Metabolism, Bone development, Translation

## Abstract

Translation of mRNA to protein is tightly regulated by transfer RNAs (tRNAs), which are subject to various chemical modifications that maintain structure, stability, and function. Deficiency of tRNA *N*^7^-methylguanosine (m^7^G) modification in patients causes a type of primordial dwarfism, but the underlying mechanism remains unknown. Here we report that the loss of m^7^G rewires cellular metabolism, leading to the pathogenesis of primordial dwarfism. Conditional deletion of the catalytic enzyme *Mettl1* or missense mutation of the scaffold protein *Wdr4* severely impaired endochondral bone formation and bone mass accrual. Mechanistically, *Mettl1* knockout decreased abundance of m^7^G-modified tRNAs and inhibited translation of mRNAs relating to cytoskeleton and Rho GTPase signaling. Meanwhile, *Mettl1* knockout enhanced cellular energy metabolism despite incompetent proliferation and osteogenic commitment. Further exploration revealed that impairment of Rho GTPase signaling upregulated the level of branched-chain amino acid transaminase 1 (BCAT1) that rewired cell metabolism and restricted intracellular α-ketoglutarate (αKG). Supplementation of αKG ameliorated the skeletal defect of *Mettl1*-deficient mice. In addition to the selective translation of metabolism-related mRNAs, we further revealed that *Mettl1* knockout globally regulated translation via integrated stress response (ISR) and mammalian target of rapamycin complex 1 (mTORC1) signaling. Restoring translation by targeting either ISR or mTORC1 aggravated bone defects of *Mettl1*-deficient mice. Overall, our study unveils a critical role of m^7^G tRNA modification in bone development by regulation of cellular metabolism and indicates suspension of translation initiation as a quality control mechanism in response to tRNA dysregulation.

## Abstract 2

Mettl1/Wdr4 mediated tRNA m7G modification regulates bone development by regulating cellular metabolism

## Graphical abstract

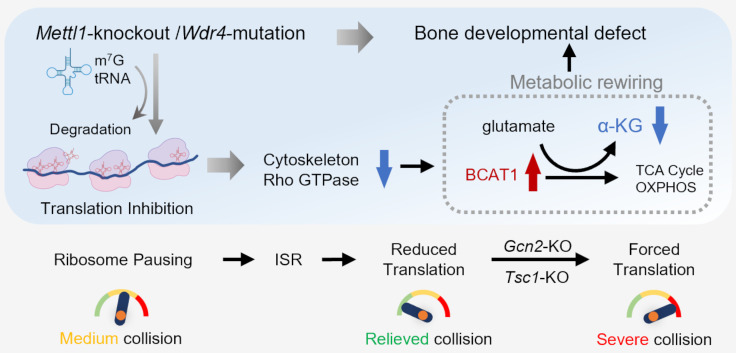

## Introduction

Eukaryotic mRNA translation to protein is an extremely complicated process that involves sequential steps of translation initiation, elongation, and termination (1). Transfer RNAs (tRNAs) act as core components of the translation machinery by recognizing codons on mRNAs and donating amino acids to form peptides. tRNAs are subject to various modifications, such as 1-methyladenosine, 5-methylcytosine, pseudouridylation, and 7-methylguanosine (2). These modifications guarantee optimal codon usage and effective protein synthesis. Loss of modifications disrupts tRNA structure, stability, and function, results in mRNA translation defects, and is implicated in developmental disorders and cancer occurrence (3, 4).

N^7^-Methylguanosine (m^7^G) is one of the most prevalent tRNA modifications and is evolutionarily conserved across prokaryotes, eukaryotes, and some archaea (5, 6). It is most frequently observed at nucleotide position 46 of the tRNA variable loop region, and meanwhile is found within rRNA, mRNA, and microRNA (6–10). In yeast, tRNA m^7^G is deposited by Trm8p/Trm82p methyltransferase (11). It protects tRNAs from rapid decay and is essential for cell growth under heat stress (12, 13). In mammals, tRNA m^7^G is catalyzed by a heterodimeric complex composed of methyltransferase-like 1 (METTL1) and WD repeat domain 4 (WDR4) (14, 15). It is required for self-renewal and differentiation of mouse embryonic stem cells (7). Homozygous missense mutation of *WDR4* is found to cause microcephalic primordial dwarfism, manifested as severe growth retardation and brain abnormalities (16). Several studies have recently revealed dysregulated tRNA m^7^G modification in cancer (17–19). However, the physiological role of m^7^G tRNA modification in organ development is unknown.

The developing limb of the mouse is an excellent model to study vertebrate organogenesis (20). The development initiates with the emergence of limb buds, followed by coordinated limb patterning and outgrowth process in which the skeletal identity is gradually established (20). Endochondral ossification then occurs, involving sequential steps of mesenchymal condensation, chondrogenesis, chondrocyte proliferation and hypertrophy, and primary ossification center formation (21, 22). Endochondral ossification contributes to longitudinal bone growth and proceeds until postnatal sexual maturity (23, 24). The involvement of morphogens (e.g., Hedgehog, FGFs, WNTs) and transcription factors (e.g., Sox9, Runx2, Sp7) in bone development has been well documented over the decades (25). Recent studies have indicated potent roles of post-transcriptional regulation in bone health (26). For example, we previously identified that METTL3-mediated mRNA *N*^6^-methyladenosine modification determined fate commitment of skeletal stem cells (27). Nevertheless, the role of tRNAs and translation regulation in the skeletal system remains largely unexplored.

Here, we show that tRNA m^7^G modification is essential to limb development. Loss of m^7^G modification severely impaired long bone development, phenocopying the primordial dwarfism. m^7^G deficiency selectively inhibited cytoskeleton and Rho GTPase signaling, which upregulated the level of branched-chain amino acid transaminase 1 (BCAT1) that rewired cellular metabolism and depleted α-ketoglutarate. We further revealed that pausing of translation initiation helped deal with tRNA-related stress. Restoring translation initiation and protein synthesis by targeting either eIF2α or 4E-BP1 exacerbated the developmental deficiency of m^7^G-deficient mice.

## Results

### Deletion of Mettl1 impairs skeletal growth.

We first explored the expression of tRNA m^7^G methyltransferase METTL1 in mouse long bone tissues. At embryonic day 16.5 (E16.5), postnatal day 0 (P0), and P14 when bone is under rapid growth, METTL1 was widely expressed in epiphyseal chondrocytes, osteoblasts, and osteocytes at the primary ossification center, and cells at the perichondrium, periosteum, and bone marrow (Supplemental Figure 1; supplemental material available online with this article; https://doi.org/10.1172/JCI177220DS1). At P28 when longitudinal growth slows, however, METTL1 expression markedly decreased and was restricted to a limited number of cells at articular cartilage and bone marrow (Supplemental Figure 1). This expression pattern led to the hypothesis that METTL1 plays a role in long bone development. To prove it, we generated mice with conditional *Mettl1* knockout driven by *Prrx1^Cre^*, which is exclusively expressed in limb bud mesenchyme from the earliest stage of bone development (Supplemental Figure 2A) (28). Immunostaining confirmed successful deletion of METTL1 in osteochondral lineage of *Prrx1^Cre^ Mettl1^fl/fl^* mice (Supplemental Figure 2, B and C). *Prrx1^Cre^ Mettl1^fl/fl^* newborn pups were viable and survived postnatally, but exhibited severe shortening of the limbs compared with *Mettl1^fl/fl^* or *Prrx1^Cre^ Mettl1^fl/+^* (hereafter, WT) littermates at birth (Supplemental Figure 2D).

**Figure 1:**
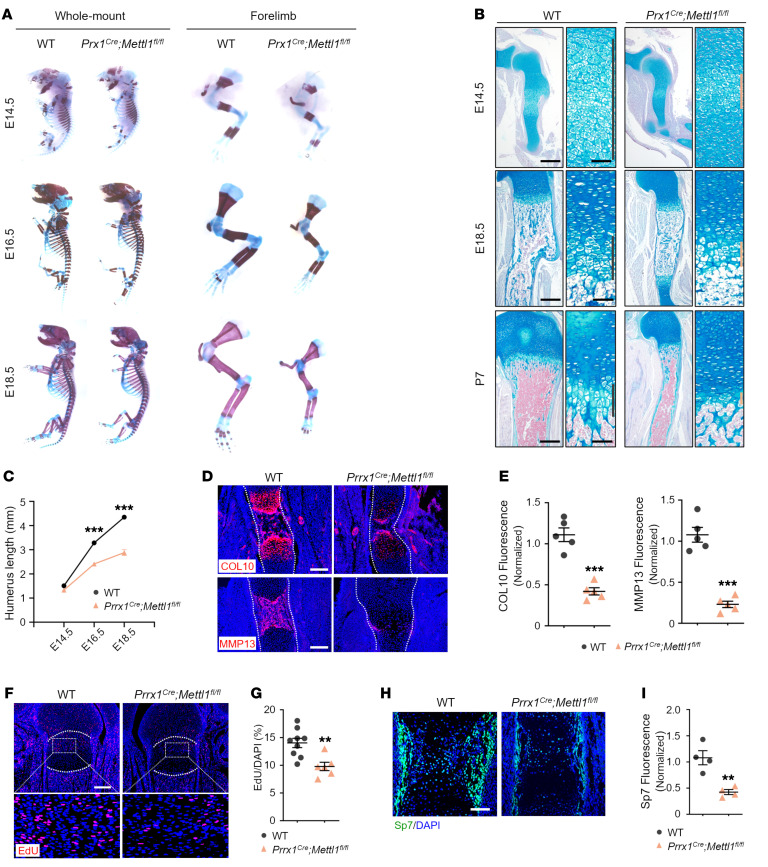
Deletion of *Mettl1* impairs skeletal growth. (**A**) Representative skeletal staining of WT and *Prrx1^Cre^ Mettl1^fl/fl^* mouse forelimbs at E14.5, E16.5, and E18.5. (**B**) Representative Alcian blue staining of WT and *Prrx1^Cre^ Mettl1^fl/fl^* mouse humerus at E14.5, E18.5, and P7. Hypertrophic zone is magnified. Scale bars: 200 μm (left) and 40 μm (right). (**C**) Quantification of humerus length at E14.5, E16.5, and E18.5. *n* = 3. (**D** and **E**) Representative immunostaining and quantification of collagen type X (Col10) and matrix metalloproteinase 13 (MMP13) of WT and *Prrx1^Cre^ Mettl1^fl/fl^* humerus at E16.5. Scale bars: 200 μm. (**F** and **G**) Representative EdU labeling and quantification of WT and *Prrx1^Cre^ Mettl1^fl/fl^* mouse femur at E16.5. Boxed areas are magnified. Scale bar: 200 μm. (**H** and **I**) Representative immunostaining and quantification of Sp7 of WT and *Prrx1^Cre^ Mettl1^fl/fl^* mouse humerus at E16.5. Scale bars: 100 μm. Data are expressed as mean ± SEM; **P < 0.01, ***P < 0.001 by 2-tailed Student’s *t* test.

To reveal the developmental defect, we first examined mice at E12.5 when mesenchymal cells initially condensed into cartilage anlagen; the digital rays were correctly patterned, and no visible difference of cartilage shape and size was found between WT and *Prrx1^Cre^ Mettl1^fl/fl^* mice (Supplemental Figure 3, A and B). The expression of SOX9 was normally induced as well (Supplemental Figure 3, C and D). At E14.5, skeletal staining showed that the stylopod and zeugopod of *Prrx1^Cre^ Mettl1^fl/fl^* mice appeared normal in length but were hypomineralized (Figure 1A). At E16.5, *Prrx1^Cre^ Mettl1^fl/fl^* mice already exhibited reduced length and hypomineralization of the humerus compared with WT mice (Figure 1A). At E18.5 and P0, a proportional shortening of all the limb skeletal elements was clearly present in *Prrx1^Cre^ Mettl1^fl/fl^* mice (Figure 1A). Quantification of humeral length indicated that the short limb phenotype started from E14.5 (Figure 1C). Therefore, METTL1 seems dispensable for initial chondrogenesis and is required for later-stage growth of the fetal bone.

Next, we histologically examined the changes of chondrocytes after *Mettl1* knockout. Alcian blue staining of the humerus showed a marked reduction of the growth plate hypertrophic zone in *Prrx1^Cre^ Mettl1^fl/fl^* mice at E14.5, E18.5, and P7 (Figure 1B). Moreover, whereas chondrocytes within the epiphysis of WT mice underwent hypertrophy and began to develop into a secondary ossification center at P7, the chondrocytes of the *Prrx1^Cre^ Mettl1^fl/fl^* mice remained in small round shape (Figure 1B). Similarly, the secondary ossification center of *Prrx1^Cre^ Mettl1^fl/fl^* mouse femora was not even formed at P21 (Supplemental Figure 4A). We further validated the defect in hypertrophy by immunostaining. The expression of collagen type X (COL10), a marker for hypertrophic chondrocytes, was barely detected (Figure 1, D and E). The expression of matrix metalloproteinase 13 (MMP13), a marker of both terminal hypertrophic chondrocytes and osteoblasts, was impaired in *Prrx1^Cre^ Mettl1^fl/fl^* mice as well (Figure 1, D and E).

Moreover, we tested the effect of *Mettl1* deletion on chondrocyte proliferation and apoptosis. The number of 5-ethynyl-2′-deoxyuridine–labeled (EdU-labeled) chondrocytes was greatly reduced in the proliferating zone of the growth plate upon *Mettl1* knockout at different time points examined (Figure 1, F and G). However, terminal deoxynucleotidyl transferase–mediated dUTP nick end labeling (TUNEL) assay did not detect visible change of chondrocyte apoptosis in *Prrx1^Cre^ Mettl1^fl/fl^* mice (Supplemental Figure 4, B and C). These results indicate that METTL1 in the limb mesenchyme is essential for chondrocyte proliferation and maturation.

### Deletion of Mettl1 reduces bone mass.

We next examined whether *Mettl1* deletion affected the osteogenic commitment and bone formation. At E16.5, Sp7-expressing osteoblast precursors were readily present in perichondrium/periosteum and primary ossification center of WT humerus, but the number was markedly reduced in *Prrx1^Cre^ Mettl1^fl/fl^* mice (Figure 1, H and I). Alcian blue/von Kossa staining of the humeral section revealed impaired matrix mineralization in *Prrx1^Cre^ Mettl1^fl/fl^* mice at E18.5 (Supplemental Figure 4D). Consistently, *Prrx1^Cre^ Mettl1^fl/fl^* mice exhibited a marked reduction of body size and femur length at P21 (Supplemental Figure 5A). The length of the growth plate proliferating and hypertrophic zone was significantly reduced (Supplemental Figure 5, B and C). Micro-CT analyses of the distal femur revealed that bone mineral density (BMD), trabecular bone volume per tissue volume (BV/TV), trabecular thickness (Tb.Th), and trabecular number (Tb.N) were decreased and trabecular bone separation (Tb.Sp) was increased in *Prrx1^Cre^ Mettl1^fl/fl^* mice (Supplemental Figure 5, D and E).

To rule out a secondary effect of impaired chondrocyte function on bone formation of *Prrx1^Cre^ Mettl1^fl/fl^* mice, we crossed *Mettl1^fl/fl^* mice to *Sp7^Cre^*, which more specifically targets osteoblastic lineages. *Sp7^Cre^ Mettl1^fl/fl^* mice were smaller than *Sp7^Cre^* mice at P21 (Supplemental Figure 6A). Histological examination and micro-CT analyses also confirmed decreased BMD, BV/TV, Tb.N, and Tb.Th and increased Tb.Sp in *Sp7^Cre^ Mettl1^fl/fl^* mice compared with *Sp7^Cre^* mice (Supplemental Figure 6, B and C).

We further isolated skeletal stem cells (SSCs) from WT and *Prrx1^Cre^ Mettl1^fl/fl^* mouse bone marrow and induced them toward osteogenic differentiation. The alkaline phosphatase (ALP) activity and calcium nodule deposition of *Prrx1^Cre^ Mettl1^fl/fl^* SSCs were weaker than those of WT SSCs (Supplemental Figure 7, A–C). Besides, the expression of osteogenesis-related markers, including *Runx2*, *Sp7*, *Alp*, *Col1a1*, and *Bglap*, was decreased in *Prrx1^Cre^ Mettl1^fl/fl^* SSCs (Supplemental Figure 7D).

### Wdr4-R215L mutation leads to bone growth defects.

**Figure 2:**
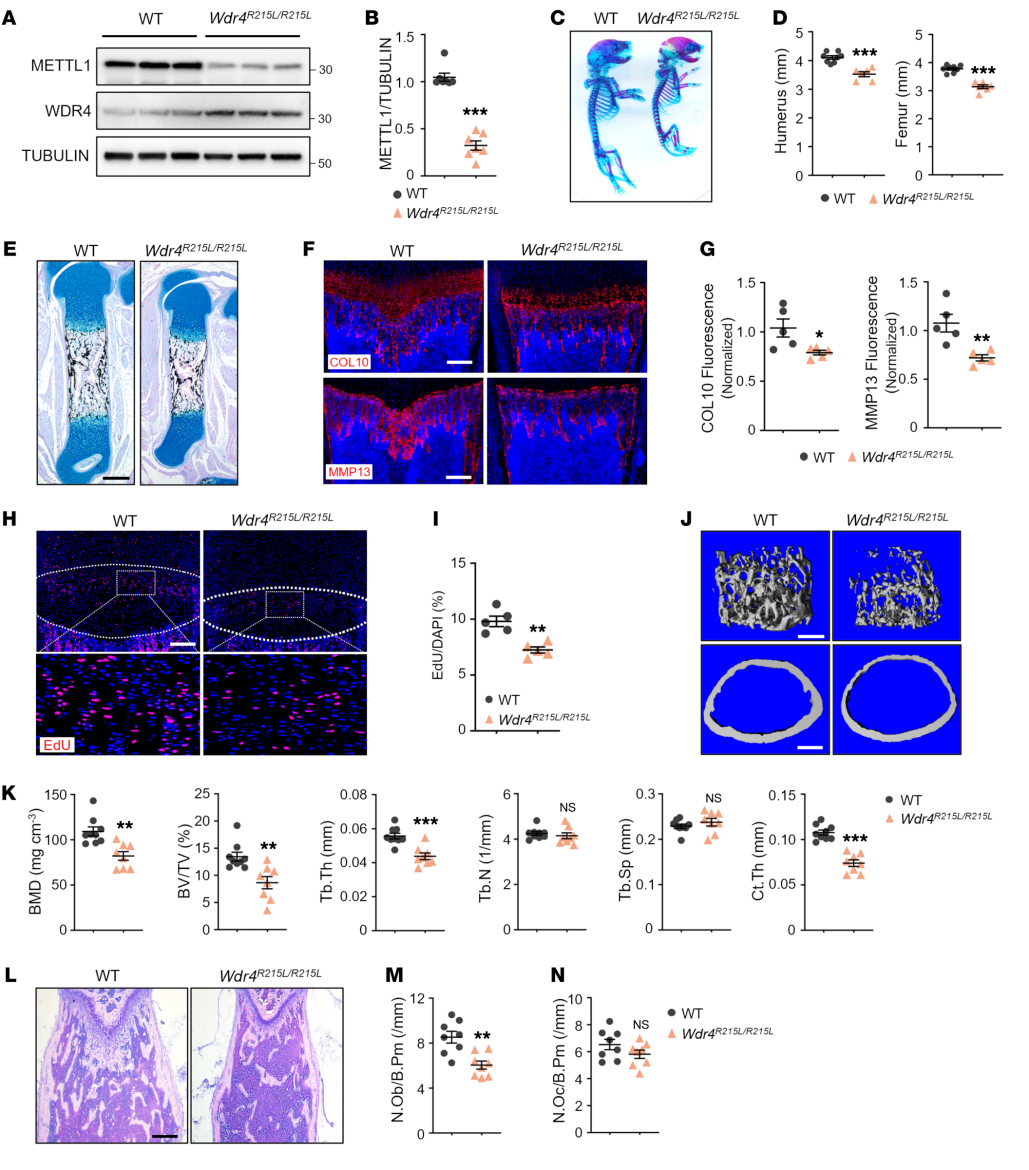
*Wdr4-R215L* mutation leads to bone growth defects. (**A** and **B**) Western blot analysis of METTL1 and WDR4 expression in WT and *Wdr4^R215L/R215L^* mouse limb tissue lysates at E16.5. *n* = 7. (**C** and **D**) Representative skeletal staining and quantification of WT and *Wdr4^R215L/R215L^* mouse limbs at P0. *n* = 8 for WT and *n* = 6 for *Wdr4^R215L/R215L^*. (**E**) Representative Alcian blue/von Kossa staining of WT and *Wdr4^R215L/R215L^* mouse femurs at P0. Scale bar: 200 μm. (**F** and **G**) Representative immunostaining and quantification of Col10 and MMP13 of WT and *Wdr4^R215L/R215L^* femora at P10. Scale bars: 200 μm. (**H** and **I**) Representative EdU labeling and quantification of WT and *Wdr4^R215L/R215L^* mouse femur at P7. Boxed areas are magnified. Scale bar: 200 μm. (**J** and **K**) Representative μCT images of femoral trabecular bone (top) and cortical bone (bottom) and related quantification from WT and *Wdr4^R215L/R215L^* mice at 6 weeks of age. BMD, bone mineral density; BV/TV, trabecular bone volume per tissue volume; Tb.Th, trabecular thickness; Tb.N, trabecular number; Tb.Sp, trabecular bone separation; Ct.Th, cortical bone thickness. Scale bars: 400 μm. *n* = 8. (**L**) Representative H&E staining of WT and *Wdr4^R215L/R215L^* mouse femur at 6 weeks of age. Scale bar: 200 μm. (**M**) Quantification of osteoblast numbers per bone perimeter (N.Ob/B.Pm). *n* = 8. (**N**) Quantification of osteoclast numbers per bone perimeter (N.Oc/B.Pm). *n* = 8. Data are expressed as mean ± SEM; *P < 0.05, **P < 0.01, ***P < 0.001 by 2-tailed Student’s *t* test.

WDR4 functions as a scaffold to support tRNA binding and METTL1 activity. Human *WDR4-R170L* mutation disrupts scaffold rigidity and decreases methyltransferase activity of METTL1 by one-third, leading to primordial dwarfism (14–16). To explore the effect of *WDR4* mutation on bone development, we introduced the *Wdr4* mutation (*R215L*, corresponding to human *WDR4-R170L*) into mice (Supplemental Figure 8A). Heterozygous *Wdr4^R215L/+^* mice were mated to obtain homozygous mutants (Supplemental Figure 8B). We verified that tRNA m^7^G modification was greatly inhibited in *Wdr4^R215L/R215L^* mice (Supplemental Figure 8C). Interestingly, the level of WDR4 was elevated in *Wdr4^R215L/R215L^* limb tissue, while that of METTL1 was markedly reduced (Figure 2, A and B, and Supplemental Figure 8D). These results suggest that loss of WDR4 scaffold rigidity reduces the stability of METTL1.

Notably, *Wdr4^R215L/R215L^* newborn pups were smaller, with reduced length of the body and the limbs, compared with WT newborn pups (Figure 2, C and D). Histological examination revealed shortening of the *Wdr4^R215L/R215L^* mouse humerus and femur compared with WT at P0 (Figure 2E). We performed immunostaining to test whether *Wdr4* mutation impairs endochondral ossification, and the results showed that Col10-positive domain and MMP13-positive domain were reduced in *Wdr4^R215L/R215L^* mice (Figure 2, F and G). Moreover, *Wdr4* mutation reduced the number of EdU-labeled growth plate chondrocytes without significant impact on apoptosis (Figure 2, H and I, and Supplemental Figure 8, E and F).

At 6 weeks of age, *Wdr4^R215L/R215L^* mice exhibited smaller body size and lighter weight, and displayed neural and behavior disorders (Supplemental Figure 9, A and B, and Supplemental Video 1). Moreover, micro-CT analyses of distal femur revealed low bone mass in *Wdr4^R215L/R215L^* mice, with a reduction in BMD, BV/TV, Tb.Th, and cortical bone thickness compared with WT (Figure 2, J and K). Histological analyses confirmed decreased bone formation and reduced number of osteoblasts in *Wdr4^R215L/R215L^* mice (Figure 2, L and M). The number of osteoclasts was comparable between WT and *Wdr4^R215L/R215L^* mice (Figure 2N and Supplemental Figure 9C). Besides, osteogenic induction of SSCs in vitro also revealed diminished ALP activity and calcium mineralization in *Wdr4^R215L/R215L^* mice (Supplemental Figure 9, D–G). Taken together, these results indicate that *Wdr4-R215L* homozygous mutation leads to bone growth defects, which further verifies the essential role of tRNA m^7^G modification in bone development.

### Mettl1 deletion erases m^7^G modification and reduces m^7^G-modified tRNA abundance.

**Figure 3:**
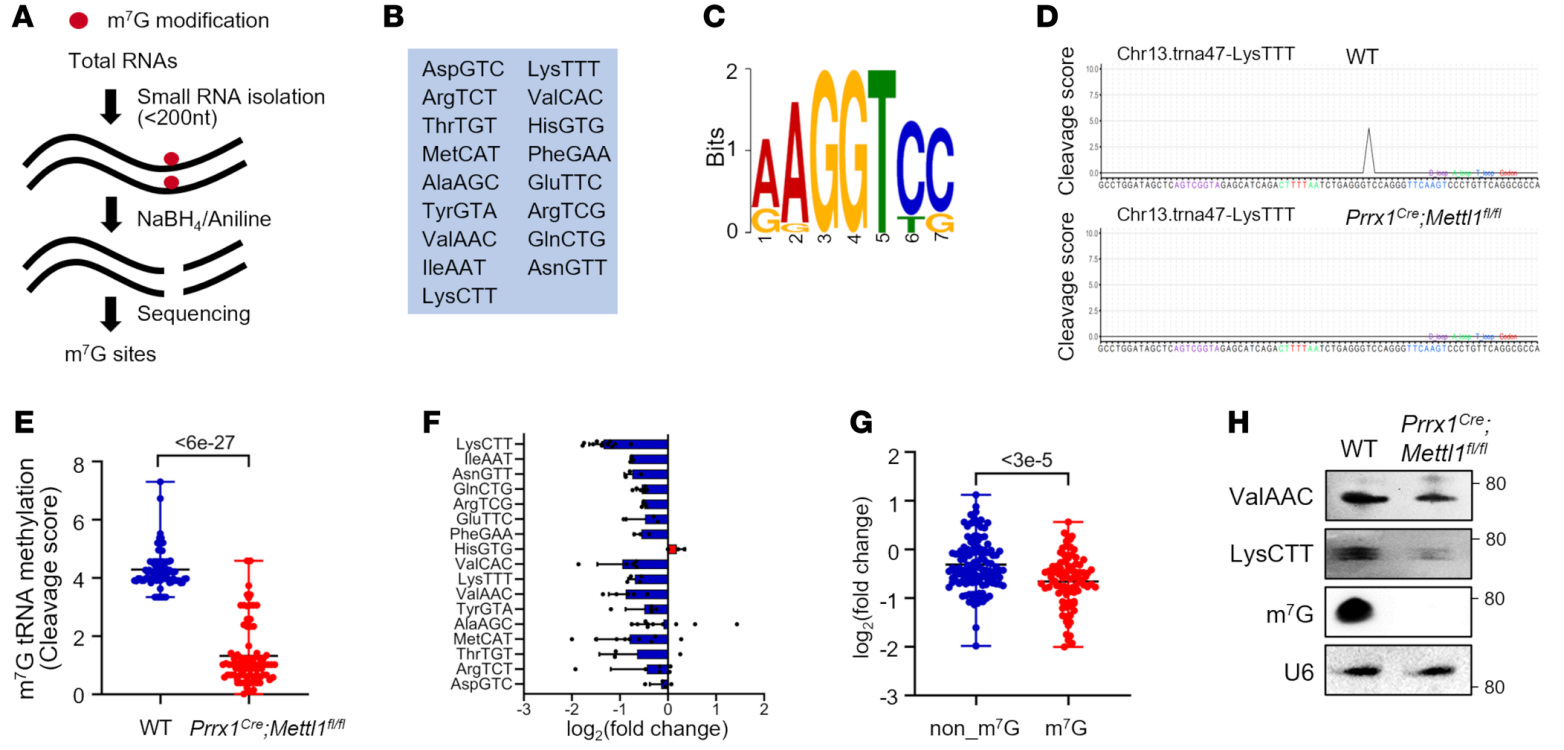
*Mettl1* deletion erases m^7^G modification and reduces m^7^G-modified tRNA abundance. (**A**) Schematic diagram of m^7^G tRNA reduction and cleavage sequencing (TRAC-Seq). (**B**) List of 17 subtypes of tRNAs with m^7^G modification identified by m^7^G TRAC-Seq in WT and *Prrx1^Cre^ Mettl1^fl/fl^* mouse limb mesenchymal progenitors at E14.5. (**C**) Sequence motif at the m^7^G sites identified by m^7^G TRAC-Seq. (**D**) Representative cleavage score of tRNA LysTTT. (**E**) Quantification of m^7^G tRNA methylation level as calculated by cleavage score. (**F**) Expression profile of the m^7^G-modified tRNAs in *Prrx1^Cre^ Mettl1^fl/fl^* mouse limb mesenchymal progenitors. The expression level of relative tRNA was calculated as the combined expression of all the tRNA genes for the same type of tRNA. (**G**) Quantification of the level of non-m^7^G-modificed tRNAs and m^7^G-modified tRNAs in *Prrx1^Cre^ Mettl1^fl/fl^* mouse limb mesenchymal progenitors. (**H**) Northern blot validation of indicated tRNAs and Northwestern validation of m^7^G modification in WT and *Prrx1^Cre^ Mettl1^fl/fl^* mouse limb mesenchymal progenitors. U6 small nuclear RNA was used as loading control. *P* values were calculated by Mann-Whitney *U* test (**E** and **G**).

To explore the underlying mechanism, we profiled the global tRNA m^7^G modification using an m^7^G tRNA reduction and cleavage sequencing (TRAC-Seq) technique (Figure 3A) (29). Seventeen m^7^G-modified tRNAs with a “RAGGU” motif were identified in limb mesenchymal progenitors (Figure 3, B and C). *Mettl1* knockout greatly decreased m^7^G tRNA methylation level as calculated by the cleavage score (Figure 3, D and E). Consequently, the abundance of m^7^G-modified tRNAs was significantly reduced and lower than that of non-m^7^G-modified tRNAs in *Prrx1^Cre^ Mettl1^fl/fl^* cells (Figure 3, F and G). Moreover, Northern blot analysis of tRNA ValAAC and LysCCT confirmed the decrease of m^7^G-modified tRNAs (Figure 3H).

### Deletion of Mettl1 impairs cytoskeleton and Rho GTPase signaling.

**Figure 4:**
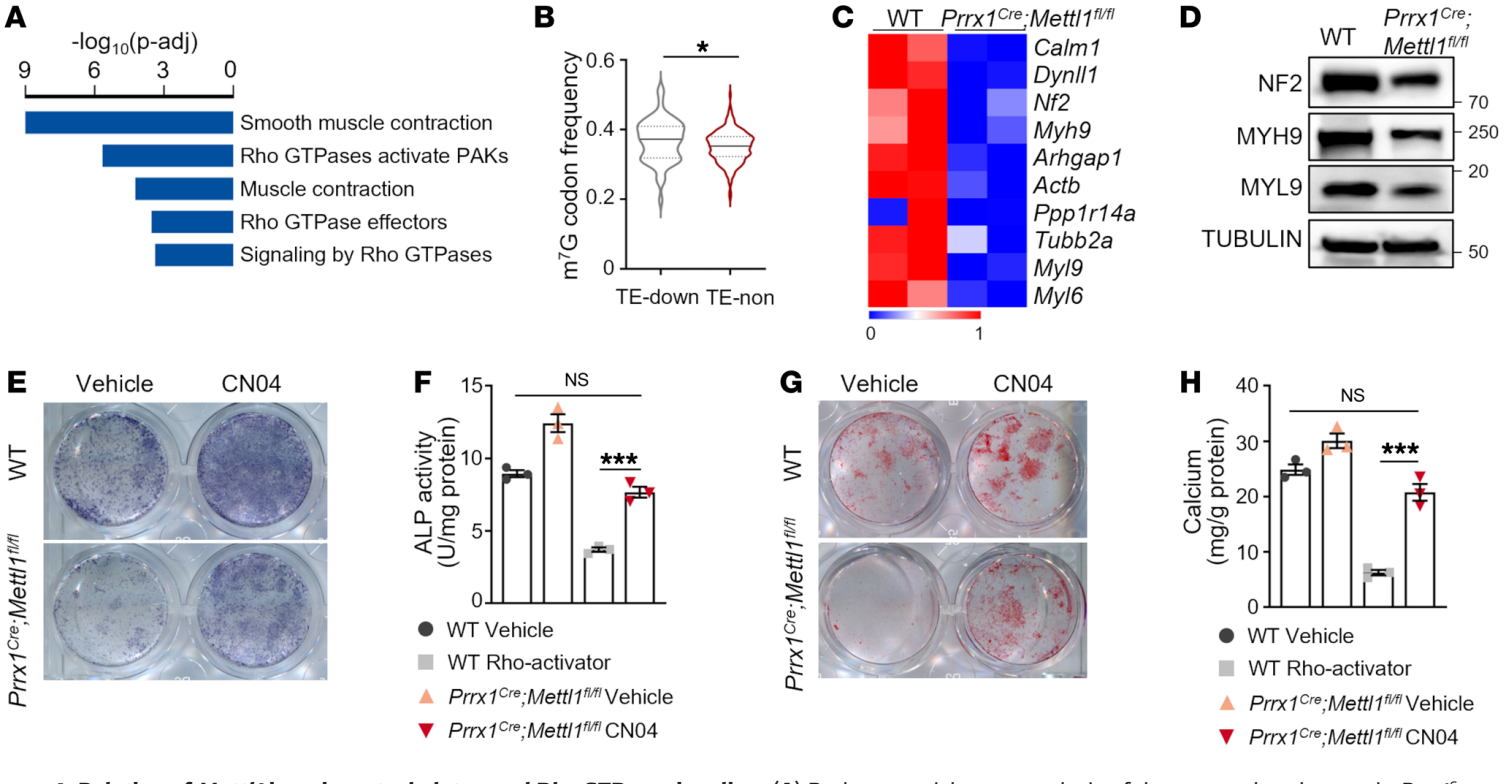
Deletion of *Mettl1* impairs cytoskeleton and Rho GTPase signaling. (**A**) Pathway enrichment analysis of down-translated genes in *Prrx1^Cre^ Mettl1^fl/fl^* limb mesenchymal progenitors at E14.5 identified by ribosome profiling. (**B**) Frequency analysis of m^7^G tRNA–decoded codons in genes with reduced translation (TE-down) and without translational change (TE-non) in *Prrx1^Cre^ Mettl1^fl/fl^* limb mesenchymal progenitors. (**C**) Heatmap of translational change of cytoskeleton-related genes. (**D**) Western blot analysis of cytoskeleton proteins in WT and *Prrx1^Cre^ Mettl1^fl/fl^* limb mesenchymal progenitors at E14.5. (**E** and **F**) Representative images and quantification of alkaline phosphatase (ALP) staining of bone marrow SSCs isolated from WT and *Prrx1^Cre^ Mettl1^fl/fl^* mice at 3 weeks of age. *n* = 3 from independent experiments. (**G** and **H**) Representative images and quantification of alizarin red S (ARS) staining of bone marrow SSCs isolated from WT and *Prrx1^Cre^ Mettl1^fl/fl^* mice at 3 weeks of age. *n* = 3 from independent experiments. Data are expressed as mean ± SEM; *P < 0.05, ***P < 0.001 by Mann-Whitney *U* test (**B**) or 1-way ANOVA with Tukey’s post hoc test (**F** and **H**).

Because *Mettl1* deletion disrupted the balance of the tRNA pool by reducing the abundance of m^7^G-modified tRNAs without major impact on non-m^7^G-modified tRNAs, mRNAs with different distribution of codons were differentially translated (7). To identify differentially translated mRNAs upon *Mettl1* deletion, we isolated limb mesenchymal cells at E14.5 and subjected them to ribosome profiling, in which the ribosome-protected mRNA fragments were collected for sequencing (Supplemental Figure 10A). A total number of 148 differentially translated mRNAs were identified, including 85 mRNAs with upregulated translation efficiency (TE-up) and 63 mRNAs with downregulated TE (TE-down) (Supplemental Figure 10B and Supplemental Table 4). Gene ontology and pathway analyses showed that TE-down mRNAs were enriched for actin cytoskeleton, muscle contraction, and Rho GTPase signaling (Figure 4A and Supplemental Figure 10C). Codon frequency analysis showed that TE-down mRNAs contained a higher percentage of codons decoded by m^7^G-modified tRNAs, compared with mRNAs without significant change of translation (TE-non), suggesting that the downregulation of cytoskeleton-related genes is directly caused by *Mettl1* deletion (Figure 4B). To test whether cytoskeletal abnormality leads to the bone defect, we first validated the translational downregulation of proteins related to the cytoskeleton. Western blot confirmed downregulated expression of cytoskeleton-related proteins, including neurofibromin 2 (NF2), myosin heavy chain 9 (MYH9), and myosin light chain 9 (MYL9), in *Prrx1^Cre^ Mettl1^fl/fl^* cells, validating the results of ribosome sequencing (Ribo-Seq) (Figure 4, C and D, and Supplemental Figure 10D). Since gene enrichment analysis suggested that the Rho GTPase signaling pathway was affected in *Mettl1*-knockout cells, and Rho GTPase is a main regulator of cytoskeleton reorganization and dynamics, we manipulated Rho GTPase signaling to reorganize the cytoskeleton and tested whether activation of Rho GTPase signaling was capable of restoring the phenotype. We treated cells with CN04, an activator that directly targets RhoA, Rac1, and Cdc42, three major Rho family proteins. The results showed that treatment of CN04 greatly promoted the ALP activity and calcium mineralization of *Prrx1^Cre^ Mettl1^fl/fl^* SSCs, suggesting a restoration of osteogenic ability (Figure 4, E–H). Thus, *Mettl1* deletion impairs cytoskeleton and Rho GTPase signaling, leading to bone growth defect.

### Deletion of Mettl1 rewires cellular respiration and energy status.

**Figure 5:**
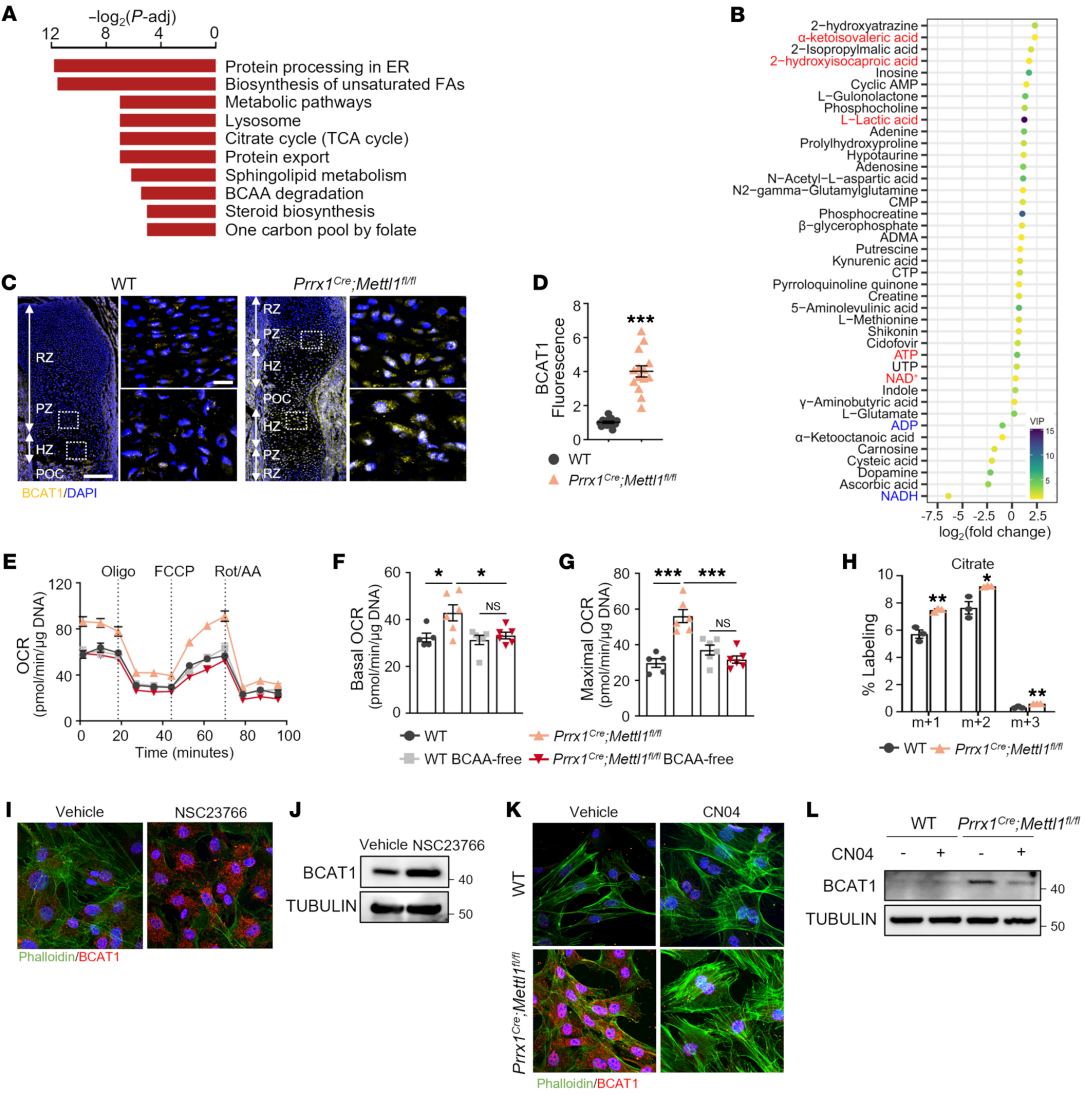
*Mettl1* knockout upregulates BCAT1 translation to support cellular respiration. (**A**) Pathway enrichment analysis of up-translated genes in *Prrx1^Cre^ Mettl1^fl/fl^* limb mesenchymal progenitors at E14.5 identified by ribosome profiling. (**B**) Differential metabolites identified in *Prrx1^Cre^ Mettl1^fl/fl^* limb mesenchymal progenitors at E14.5 by untargeted metabolomics. (**C** and **D**) Immunostaining and quantification of BCAT1 in WT and *Prrx1^Cre^ Mettl1^fl/fl^* mouse humerus at E16.5. Boxed areas are magnified. RZ, resting zone; PZ, proliferating zone; HZ, hypertrophic zone; POC, primary ossification center. *n* = 14. Scale bars: 200 μm (left) and 20 μm (right). (**E**–**G**) Representative graph of oxygen consumption rate (OCR) and quantification at baseline and with indicated drugs. Oligo, oligomycin; FCCP, carbonyl cyanide-4 (trifluoromethoxy)phenylhydrazone; Rot/AA, rotenone and antimycin A. *n* = 5 (WT) or 6 (other groups). (**H**) Abundance of ^13^C-labeled citrate in WT and *Prrx1^Cre^ Mettl1^fl/fl^* cells. *n* = 3. (**I**) Representative immunostaining of BCAT1 expression in vehicle- and NSC23766-treated chondrocytes. (**J**) Western blot analysis of BCAT1 expression in vehicle- and NSC23766-treated chondrocytes. (**K**) Representative immunostaining of BCAT1 expression in vehicle- and CN04-treated WT and *Prrx1^Cre^ Mettl1^fl/fl^* chondrocytes. Magnification, 800 × (**I** and **K**) (**L**) Western blot analysis of BCAT1 expression in vehicle- and CN04-treated WT and *Prrx1^Cre^ Mettl1^fl/fl^* chondrocytes. Data are expressed as mean ± SEM; *P < 0.05, **P < 0.01, ***P < 0.001 by 2-tailed Student’s *t* test (**D** and **H**) or by 1-way ANOVA with Tukey’s post hoc test (**F** and **G**).

Besides the downregulation of cytoskeleton-related proteins, we found, surprisingly, that TE-up mRNAs were enriched for metabolic processes and signaling in fatty acid synthesis, TCA cycle, branched-chain amino acid (BCAA) degradation, and one-carbon metabolism upon *Mettl1* deletion (Figure 5A and Supplemental Figure 10E). This metabolic active state is intuitively contradictory to the phenotype that *Mettl1*-deficient cells were incapable of proliferation and differentiation. Therefore, we asked why and how *Mettl1* knockout leads to changes of cell metabolism, and whether this metabolic active state also results in bone developmental defect.

We first verified the metabolic change and identified differential metabolites by performing untargeted metabolomic profiling of the limb mesenchymal progenitors (Figure 5B and Supplemental Figure 11A). We set a stringent statistical threshold (orthogonal partial least-squares discriminant analysis [OPLS-DA] variable importance in the projection [VIP] > 1 and *P* < 0.05) and identified 41 differential metabolites (Figure 5B and Supplemental Figure 11A). Pathway analysis showed plenty of changes related to neural system, such as neuroactive ligand-receptor interaction, Parkinson disease, synaptic vesicle cycle, and GABAergic synapse, reminiscent of the neurological abnormality in primordial dwarfism (Supplemental Figure 11B). Notably, metabolites were enriched for energy metabolism, including oxidative phosphorylation, thermogenesis, and hypoxia-inducible factor 1 (HIF1) signaling (Supplemental Figure 11B). When interrogating the altered metabolites, we found increase of ATP and phosphocreatine and reduction of ADP in *Prrx1^Cre^ Mettl1^fl/fl^* cells. The level of oxidized nicotinamide adenine dinucleotide (NAD^+^) was increased, whereas the reduced form of NAD (NADH) was the most decreased metabolite in *Prrx1^Cre^ Mettl1^fl/fl^* cells (Figure 5B). Besides, the intracellular content of lactate was higher, indicating increase of glycolysis (Figure 5B).

We further interrogated the cellular respiration in *Mettl1*-deficient cells. In agreement with the findings above, intracellular steady-state levels of ATP were higher in *Prrx1^Cre^ Mettl1^fl/fl^* limb mesenchymal progenitors (123.76% ± 6.45%) and chondrocytes (176.70% ± 5.24%) (Supplemental Figure 11, C and D). The energy sensor phospho-AMPK/AMPK ratio was comparable in WT versus *Prrx1^Cre^ Mettl1^fl/fl^* cells (Supplemental Figure 11E). The number of mitochondria was not significantly changed, whereas the mitochondrial ROS production was slightly increased (Supplemental Figure 11, F and G). Though the basal O_2_ consumption rate (OCR) was not significantly changed (*P* = 0.073), the maximal OCR was elevated in *Prrx1^Cre^ Mettl1^fl/fl^* cells (Supplemental Figure 11, H–J).

As lactate was increased in *Prrx1^Cre^ Mettl1^fl/fl^* cells, we then interrogated the non-oxidative glycolysis. A higher rate of lactate secretion was observed in *Prrx1^Cre^ Mettl1^fl/fl^* cells (Supplemental Figure 11K). Extracellular acidification rate measured by Seahorse assays also demonstrated a higher rate of glycolysis and augmented glycolytic capacity in *Mettl1*-deficient cells (Supplemental Figure 11, L–N). Taken together, these results indicate that loss of *Mettl1* enhanced both the mitochondrial oxidative phosphorylation and aerobic glycolysis.

### Deletion of Mettl1 upregulates BCAT1 that rewires cellular metabolism.

Hyperactivated energy production following *Mettl1* deletion requires enhanced input of nutrients to fuel the TCA cycle. Combined analysis of ribosome profiling and metabolomic data identified changes that converged on BCAA catabolism (Figure 5, A and B). The level of branched-chain amino acid transaminase 1 (*Bcat1*) was greatly increased in humeral chondrocytes of *Prrx1^Cre^ Mettl1^fl/fl^* mice (Figure 5, C and D). Western blot validated elevated METTL1 level upon *Mettl1* deletion (Supplemental Figure 12A).

BCAT1 catalyzes the first step of BCAA catabolism by deaminating BCAA to form branched-chain keto-acid (BCKA) (30). Consistently, the levels of α-ketoisovaleric acid and 2-hydroxyisocaproic acid, derivatives from the breakdown of valine and leucine, respectively, were increased in *Prrx1^Cre^ Mettl1^fl/fl^* cells (Figure 5B). To evaluate the contribution of BCAA to cellular respiration, limb mesenchymal progenitors were subjected to Seahorse assays in basal medium supplemented with BCAA or deprived of BCAA. The OCR of *Prrx1^Cre^ Mettl1^fl/fl^* cells is higher than that of WT cells when cultured in basal medium containing BCAA. Under BCAA deprivation conditions, both the basal OCR and maximal OCR of *Prrx1^Cre^ Mettl1^fl/fl^* cells were greatly reduced, with no significant difference from those of WT cells (Figure 5, E–G). Moreover, metabolic flux analysis using stable isotope–labeled [^13^C,^15^N]l-leucine revealed that the contribution of [^13^C,^15^N]l-leucine to TCA cycle intermediates was greatly increased in *Prrx1^Cre^ Mettl1^fl/fl^* cells (Figure 5H and Supplemental Figure 12B). These results indicate that BCAT1-mediated BCAA degradation supports cellular respiration upon *Mettl1* deletion.

Moreover, we examined the contribution of glucose to energy production upon *Mettl1* deletion. Despite an increase of glycolysis, glucose uptake was diminished in *Mettl1*-deficient cells (Supplemental Figure 13A). Metabolic flux analysis using stable isotope–labeled [U-^13^C]glucose revealed that the amounts of ^13^C-labeled glycolytic intermediates were slightly increased in *Prrx1^Cre^ Mettl1^fl/fl^* cells, whereas the contribution of [U-^13^C]glucose to TCA cycle intermediates was greatly reduced (Supplemental Figure 13, B and C). Thus, these findings negated glucose as a major contributor to cellular respiration in *Mettl1*-deleted cells.

### Cytoskeletal alteration regulates BCAT1 level.

We next explored why translation of mRNAs relating to cell metabolism was increased after *Mettl1* knockout. We first performed codon frequency analysis, but no significant difference relating to percentage of codons decoded by m^7^G-modified tRNAs was found between TE-up mRNAs and TE-non mRNAs, indicating that translation of TE-up mRNAs was not directly regulated by m^7^G tRNA–decoded codons (Supplemental Figure 14). Thus, we reason that the upregulation of metabolism is not directly mediated by change of m^7^G tRNA abundance, and is instead secondary to other signaling.

Studies have reported that cytoskeleton dynamics are closely associated with cellular metabolic state, and a previous report identified that numerous cytoskeletal proteins bind to BCAT1 via immunoprecipitation–mass spectrometry, supporting an interaction between cytoskeletal proteins and BCAT1 (31–33). Therefore, we explored whether BCAT1 levels were regulated by cytoskeleton. We manipulated Rho GTPase signaling to reorganize the cytoskeleton. Interestingly, we found that inhibiting Rho GTPase with NSC23766 trihydrochloride impaired cytoskeleton and upregulated BCAT1 levels in WT cells, as shown by cell immunofluorescence and Western blot (Figure 5, I and J). Conversely, activating Rho GTPase with CN04 reshaped cytoskeleton and led to lower BCAT1 levels in *Prrx1^Cre^ Mettl1^fl/fl^* cells (Figure 5, K and L). Therefore, we propose that knockout of *Mettl1* affects BCAT1 levels via its action on cytoskeleton.

### BCAT1 depletes α-ketoglutarate and its supplementation promotes long bone growth of Prrx1^Cre^ Mettl1^fl/fl^ mice.

**Figure 6:**
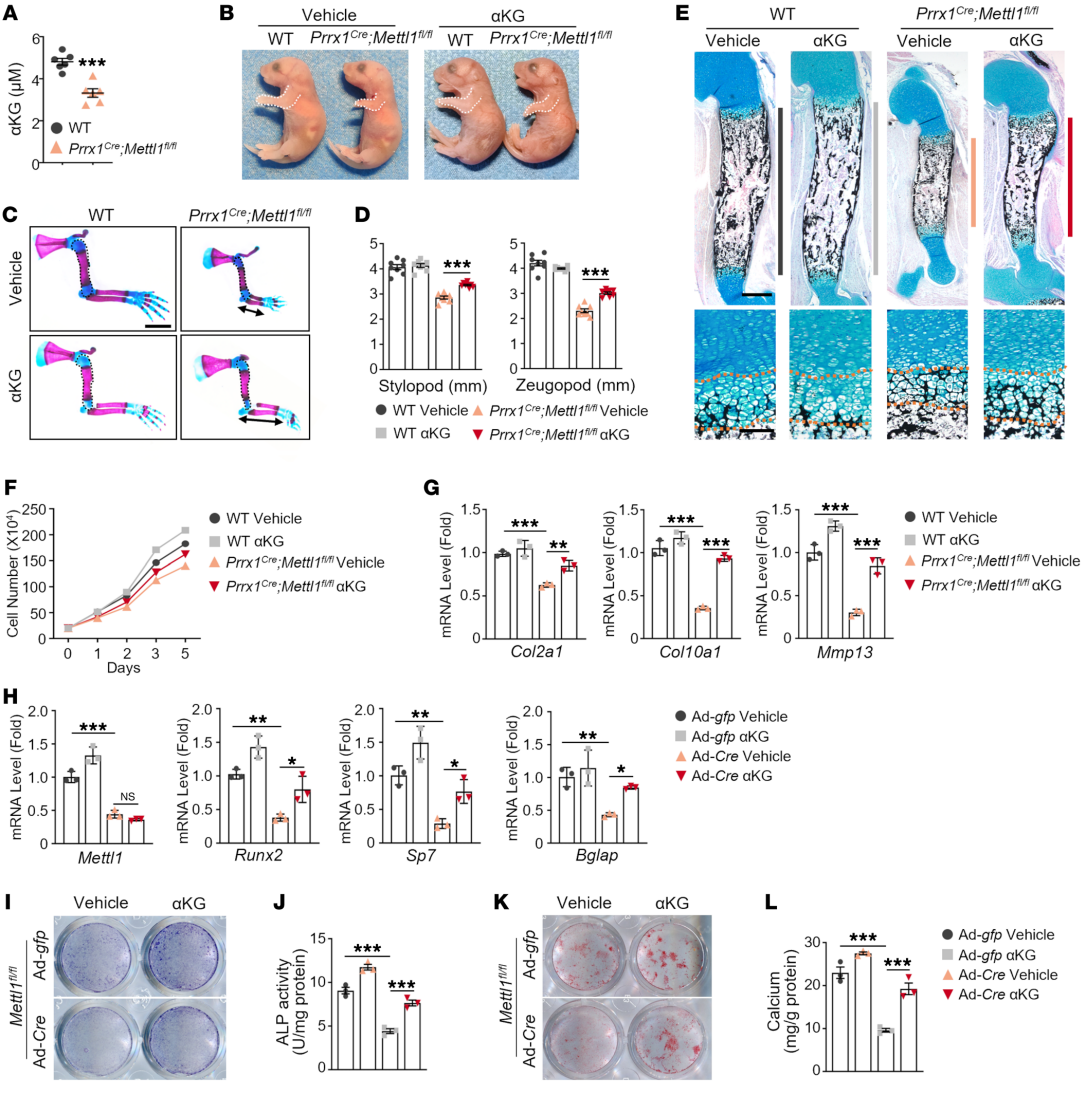
Supplementation of αKG ameliorates the skeletal defects of *Prrx1^Cre^ Mettl1^fl/fl^* mice. (**A**) Measurement of intracellular αKG level in WT and *Prrx1^Cre^ Mettl1^fl/fl^* limb mesenchymal progenitors. *n* = 6. (**B**) Representative images of newborn WT and *Prrx1^Cre^ Mettl1^fl/fl^* mice treated with vehicle or αKG. (**C**) Representative skeletal staining of WT and *Prrx1^Cre^ Mettl1^fl/fl^* mouse forelimbs at P0, treated with vehicle or αKG. Scale bar: 2 mm. (**D**) Quantification of forelimb stylopod and zeugopod length. *n* = 8. (**E**) Representative Alcian blue staining of WT and *Prrx1^Cre^ Mettl1^fl/fl^* mouse humerus at P0, treated with vehicle or αKG. Magnified images show growth plate hypertrophic zone of the proximal humerus. Scale bars: 200 μm (top) and 50 μm (bottom). (**F**) Growth curve of WT and *Prrx1^Cre^ Mettl1^fl/fl^* chondrocytes, treated with vehicle or αKG. *n* = 3. (**G**) Quantitative reverse transcriptase PCR (qRT-PCR) analyses of the expression of *Col2a1*, *Col10a1*, and *Mmp13* in WT and *Prrx1^Cre^ Mettl1^fl/fl^* chondrocytes, treated with vehicle or αKG. *n* = 3 from independent experiments. (**H**) qRT-PCR analyses of the expression of *Mettl1*, *Runx2*, *Sp7*, and *Bglap* in Ad-*gfp*– and Ad-*Cre*–infected *Mettl1^fl/fl^* SSCs, treated with vehicle or αKG. *n* = 3 from independent experiments. (**I** and **J**) Representative images and quantification of ALP staining of WT and *Prrx1^Cre^ Mettl1^fl/fl^* SSCs, treated with vehicle or αKG. *n* = 3 from independent experiments. (**K** and **L**) Representative images and quantification of ARS staining of WT and *Prrx1^Cre^ Mettl1^fl/fl^* SSCs, treated with vehicle or αKG. *n* = 3 from independent experiments. Data are expressed as mean ± SEM; *P < 0.05, **P < 0.01, ***P < 0.001 by 1-way ANOVA with Tukey’s post hoc test.

We next explored the effect of upregulated cellular energy metabolism on bone development. As BCAT1 transfers α-amino groups from BCAA to α-ketoglutarate (αKG) to form glutamate, we examined the changes. In fact, we found that the intracellular level of αKG was markedly reduced and the concentration of glutamate in culture medium was sharply increased upon *Mettl1* deletion (Figure 6A and Supplemental Figure 15A). To test whether reduced αKG led to the skeletal defect of *Prrx1^Cre^ Mettl1^fl/fl^* mice, pregnant mice were supplemented with 1% αKG in drinking water since E12.5 (Supplemental Figure 15B). The level of αKG was greatly increased in cartilage tissue of *Prrx1^Cre^ Mettl1^fl/fl^* mice after αKG supplementation (Supplemental Figure 15C). *Prrx1^Cre^ Mettl1^fl/fl^* newborn pups exhibited longer forelimbs after αKG supplement (Figure 6B). Skeletal staining showed that αKG supplementation greatly increased the length of stylopod and zeugopod (Figure 6, C and D). Histological analysis of the humerus revealed that the length of the growth plate hypertrophic zone was markedly increased in αKG-treated *Prrx1^Cre^ Mettl1^fl/fl^* mice, suggesting improved endochondral bone formation (Figure 6E). Moreover, αKG improved the proliferation of primary chondrocytes isolated from *Prrx1^Cre^ Mettl1^fl/fl^* mice (Figure 6F). The expression of the chondrocyte hypertrophic markers *Col10* and *Mmp13* was increased in *Prrx1^Cre^ Mettl1^fl/fl^* mice when supplemented with αKG (Figure 6G). Additionally, to investigate whether αKG promotes osteogenic differentiation, we isolated SSCs from *Mettl1^fl/fl^* mice and performed *Mettl1* knockout via Adeno-*Cre* transfection. The expression of osteogenesis-related genes including *Runx2*, *Sp7*, and *Bglap* was increased in *Mettl1*-deficient SSCs after αKG treatment (Figure 6H). ALP staining and alizarin red S staining revealed that αKG promoted mineralization and calcium deposition of *Mettl1*-deficient SSCs (Figure 6, I–L). Therefore, upregulated BCAT1 expression disrupts αKG homeostasis, and restoring αKG could alleviate the skeletal defect of *Prrx1^Cre^ Mettl1^fl/fl^* mice.

### Promoting translation initiation by inhibiting the integrated stress response aggravates the skeletal disorders of Mettl1-deficient mice.

**Figure 7:**
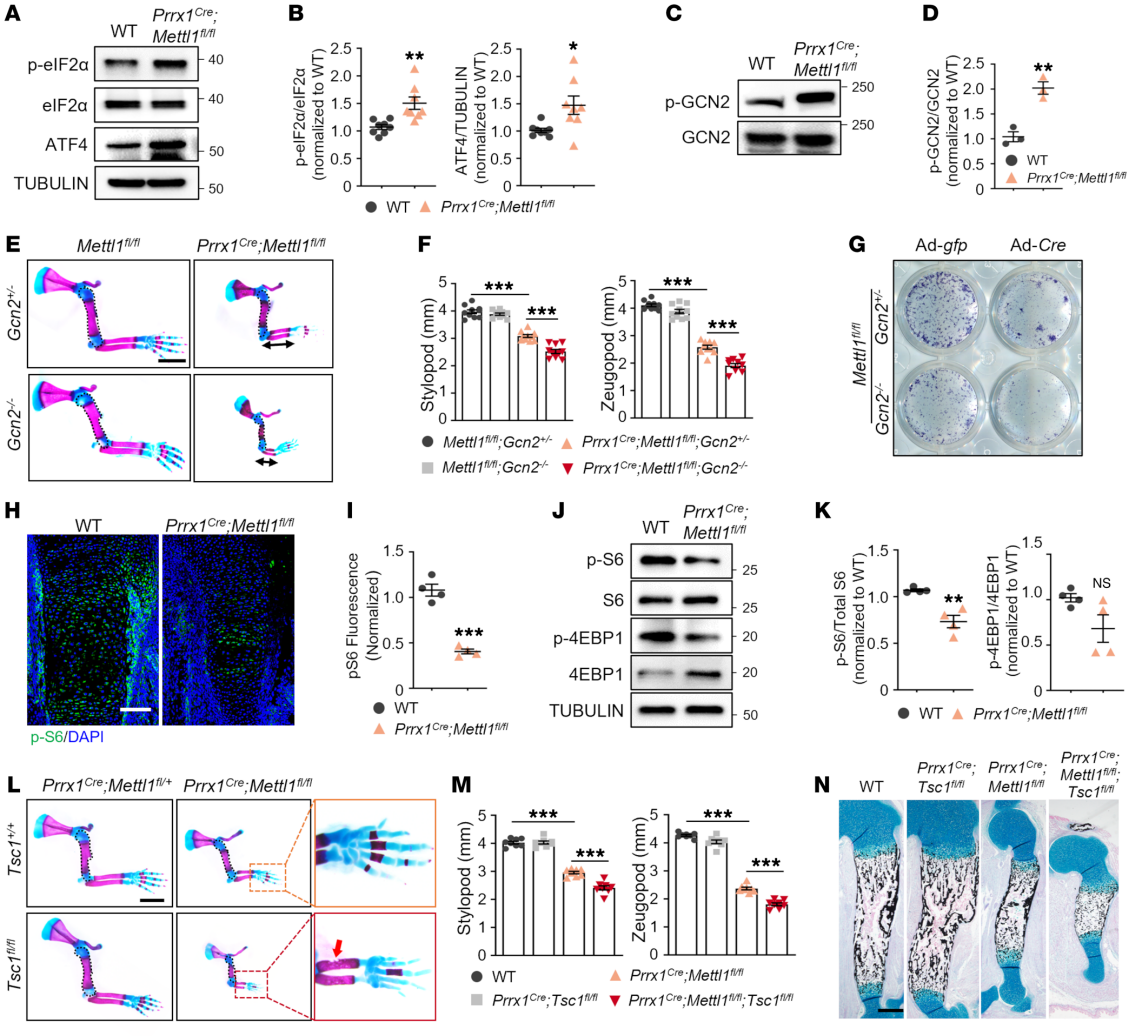
Promoting translation initiation aggravates the skeletal disorders of *Mettl1*-deficient mice. (**A** and **B**) Western blot analysis and quantification of phospho-eIF2α, eIF2α, and ATF4 in WT and *Prrx1^Cre^ Mettl1^fl/fl^* forelimb tissue lysates at E14.5. *n* = 8. (**C** and **D**) Western blot analysis and quantification of phospho-GCN2 and GCN2 levels in WT and *Prrx1^Cre^ Mettl1^fl/fl^* limb mesenchymal progenitors. *n* = 3. (**E**) Representative skeletal staining of *Mettl1^fl/fl^* and *Prrx1^Cre^ Mettl1^fl/fl^* mouse forelimbs in *Gcn2^+/–^* or *Gcn2^–/–^* background at P0. Scale bar: 2 mm. (**F**) Quantification of forelimb stylopod and zeugopod length of *Mettl1^fl/fl^* and *Prrx1^Cre^ Mettl1^fl/fl^* mice in *Gcn2^+/–^* or *Gcn2^–/–^* background. *n* = 10. (**G**) Representative images of ALP staining of bone marrow SSCs isolated from *Mettl1^fl/fl^ Gcn2^+/–^* and *Mettl1^fl/fl^ Gcn2^–/–^* mice at 4 weeks of age, and infected with Ad-*gfp* or Ad-*Cre*. (**H** and **I**) Representative immunostaining and quantification of phospho-S6 in WT and *Prrx1^Cre^ Mettl1^fl/fl^* mouse humerus at E14.5. Scale bar: 100 μm. (**J** and **K**) Western blot analysis and quantification of phospho-S6, S6, phospho–4E-BP1, and 4E-BP1 levels in WT and *Prrx1^Cre^ Mettl1^fl/fl^* mouse mesenchymal progenitors. (**L**) Representative skeletal staining of *Prrx1^Cre^ Mettl1^fl/+^* and *Prrx1^Cre^ Mettl1^fl/fl^* mouse forelimbs in *Tsc1^+/+^* or *Tsc1^fl/fl^* background at P0. Boxed areas are magnified. Arrow indicates cortical bone defect. Scale bar: 2 mm. (**M**) Quantification of forelimb stylopod and zeugopod length in WT, *Prrx1^Cre^ Tsc1^fl/fl^*, *Prrx1^Cre^ Mettl1^fl/fl^*, and *Prrx1^Cre^ Mettl1^fl/fl^ Tsc1^fl/fl^* mice at P0. *n* = 8. (**N**) Representative Alcian blue/von Kossa staining of WT, *Prrx1^Cre^ Tsc1^fl/fl^*, *Prrx1^Cre^ Mettl1^fl/fl^*, and *Prrx1^Cre^ Mettl1^fl/fl^ Tsc1^fl/fl^* mouse humerus at P0. Scale bar: 200 μm. Data are expressed as mean ± SEM; *P < 0.05, **P < 0.01, ***P < 0.001 by 2-tailed Student’s *t* test (**B**, **D**, **I**, and **K**) or 1-way ANOVA with Tukey’s post hoc test (**F** and **M**).

Previous study has shown that *Mettl1* deletion led to ribosome pause during translation elongation, and as translation initiation and elongation are highly coordinated, we further explored whether *Mettl1* affects translation initiation. Indeed, we found that phosphorylation of eukaryotic translation initiation factor 2α (eIF2α) was increased, indicating inhibition of translation initiation (Figure 7, A and B) (34, 35). We further found that *Mettl1* knockout greatly upregulated the autophosphorylation of general control nonderepressible 2 (GCN2), instead of PKR-like endoplasmic reticulum kinase (PERK) or double-stranded RNA–dependent protein kinase (PKR), which subsequently phosphorylated eIF2α and upregulated the level of activating transcription factor 4 (ATF4) (Figure 7, C and D, and Supplemental Figure 16, A and B). As GCN2 is activated in response to ribosome stalling, these results indicate that translation stress evoked by *Mettl1* deletion relays to GCN2 that phosphorylates eIF2α and activates the integrated stress response (ISR).

Inhibition of ISR was reported to alleviate cognitive and neurodegenerative disorders (34–37). To test whether inhibiting ISR could restore the skeletal development of *Prrx1^Cre^ Mettl1^fl/fl^* mice, we deleted *Gcn2* on the *Prrx1^Cre^ Mettl1^fl/fl^* background. Whole-mount skeletal staining showed no visible difference between *Gcn2^–/–^* and WT mice at P0. Nevertheless, *Prrx1^Cre^ Mettl1^fl/fl^ Gcn2^–/–^* mice showed more severe shortening of the forelimbs compared with *Prrx1^Cre^ Mettl1^fl/fl^ Gcn2^+/–^* mice (Figure 7, E and F).

Moreover, we isolated bone marrow SSCs of *Mettl1^fl/fl^ Gcn2^+/–^* and *Mettl1^fl/fl^ Gcn2^–/–^* mice, and infected them with *Cre-* or *gfp*-expressing adenoviruses. Deletion of *Gcn2* abrogated ISR and promoted translation initiation, as shown by increased incorporation of puromycin into nascent peptides (Supplemental Figure 16C). When induced toward osteogenic differentiation, Ad-*Cre* *Mettl1^fl/fl^ Gcn2^–/–^* SSCs exhibited much lower ALP activity than Ad-*Cre* *Mettl1^fl/fl^ Gcn2^+/–^* SSCs (Figure 7G). Therefore, removal of *Gcn2* abrogates ISR and restores protein synthesis, but aggravates skeletal disorders of *Prrx1^Cre^ Mettl1^fl/fl^* mice.

### Promoting translation by activating mTORC1 signaling exacerbates skeletal defect of Mettl1-deficient mice.

Apart from ISR, mammalian target of rapamycin complex 1 (mTORC1) is another central hub that regulates translation (38). It promotes translation rates by phosphorylating ribosome S6 (S6) and eIF4E-binding protein 1 (4E-BP1), which is different and independent from the ISR. Immunostaining of the humerus revealed marked reduction of phospho-S6 in *Prrx1^Cre^ Mettl1^fl/fl^* mice compared with WT (Figure 7, H and I). Western blot showed decreased levels of phospho-S6 and phospho–4E-BP1 upon *Mettl1* deletion (Figure 7, J and K). Interestingly, the expression of 4E-BP1 was elevated in *Prrx1^Cre^ Mettl1^fl/fl^* cells (Figure 7, J and K).

Next, we sought to genetically activate mTORC1 by deleting its negative regulator tuberous sclerosis complex 1 (*Tsc1*) in *Prrx1^Cre^ Mettl1^fl/fl^* mice. *Prrx1^Cre^ Mettl1^fl/fl^ Tsc1^fl/fl^* mice showed a much higher level of phospho-S6 in humeral growth plate chondrocytes, indicating upregulated mTORC1 activity (Supplemental Figure 17A). Western blot analysis confirmed increased phosphorylation of S6 and 4E-BP1 upon *Tsc1* deletion in *Mettl1*-deficient cells (Supplemental Figure 17B). Nascent peptide synthesis was partially restored after *Tsc1* deletion as well (Supplemental Figure 17C). Both gross observation and whole-mount skeletal staining showed no obvious difference between *Prrx1^Cre^ Tsc1^fl/fl^* and WT (*Prrx1^Cre^ Mettl1^fl/+^* or *Mettl1^fl/fl^*) newborn pups (Figure 7L and Supplemental Figure 17D). Remarkably, *Tsc1* deletion exacerbated the skeletal defect of *Prrx1^Cre^ Mettl1^fl/fl^* mice. The length of the stylopod and zeugopod was further shortened, and the autopod (metacarpals and phalanges) formation was more severely retarded in *Prrx1^Cre^ Mettl1^fl/fl^ Tsc1^fl/fl^* mice (Figure 7, L and M). Alcian blue/von Kossa staining of the humeral section confirmed a further shortened and hypocalcified phenotype (Figure 7N). We further cultured bone marrow SSCs of *Mettl1^fl/fl^ Tsc1^fl/+^* and *Mettl1^fl/fl^ Tsc1^fl/fl^* mice and infected them with Ad-*Cre* or Ad-*gfp*. We also observed lower ALP activity in Ad-*Cre Mettl1^fl/fl^ Tsc1^fl/fl^* SSCs as compared with Ad-*Cre Mettl1^fl/fl^ Tsc1^fl/+^* SSCs after osteogenic induction (Supplemental Figure 17E).

## Discussion

Translation is a highly complicated process that requires tight regulation (1). Disturbance of any component of the translation machinery, including ribosome, mRNA, tRNA, and the regulating factors, could directly affect the rate and amount of protein synthesis (39). Therefore, translational regulation plays an essential role in organ development and disease occurrence. Several studies have highlighted how regulation of translation affects bone health. For example, mTORC1, a dominating regulator of translation, is indispensable for bone development (40). Inhibition of mTOR signaling impedes endochondral bone formation and leads to dwarfism (41, 42). Ribosome protein also controls limb development. Haploinsufficiency of ribosome protein S6 upregulates 4E-BP1 and leads to defective forelimb patterning (43). Nevertheless, the role of tRNA in the skeletal system remains unknown. In this study, for the first time to our knowledge, we linked tRNA biology to the skeletal system. Using *Wdr4^R215L/R215L^* and *Prrx1^Cre^ Mettl1^fl/fl^* mouse models, we unambiguously revealed that METTL1/WDR4-mediated tRNA m^7^G modification is essential to endochondral bone formation and bone mass accrual. METTL1 performs a catalytic function, and we found that *Mettl1* deletion impaired proliferation and osteochondral differentiation, resulting in retarded and hypoplastic formation of cartilage and bone, resembling the brachypodism phenotype. WDR4 functions as a scaffold for METTL1 and tRNA binding, and *WDR4-R170L* homozygous mutation causes microcephalic primordial dwarfism (16). Recent structural analysis showed that R170L (corresponding to mouse R215L) disrupts the intramolecular interaction within WDR4 itself and reduces the methyltransferase activity of METTL1 by one-third (15). Consistently, phenotype analysis showed less severe bone defect of *Wdr4^R215L/R215L^* mice as compared with *Prrx1^Cre^ Mettl1^fl/fl^* mice, indicating a dose effect of m^7^G modification on bone development. Besides, we found that METTL1 protein level was reduced in *Wdr4^R215L/R215L^* limb tissue, indicating activation of a METTL1 degradation mechanism when WDR4 structure is impaired, which requires further studies.

*Mettl1* deletion in mouse embryonic stem cells reduced the abundance of m^7^G-modified tRNAs and impaired codon recognition during translation, leading to ribosome pause and selective mRNA translation (7). In this study, we verified that *Mettl1* deletion or *Wdr4* mutation in osteochondral lineage reduced m^7^G-modified tRNA pools. Using Ribo-Seq, we found that *Mettl1* deletion reduced the translational efficiency of cytoskeleton-related proteins and affected Rho GTPase signaling. Activating Rho GTPase signaling greatly promoted the osteogenesis of *Prrx1^Cre^ Mettl1^fl/fl^* SSCs. However, as the downstream effect exerted by Rho GTPase signaling is diverse and overactivated Rho GTPase contributes to cancer progression, manipulating Rho GTPase signaling to rescue skeletal development requires caution (44). A more specific downstream target is expected.

Surprisingly, we found that translation of mRNAs related to cell metabolism was upregulated after *Mettl1* deletion, which resulted in enhanced cellular respiration despite compromised cell proliferation and differentiation. More importantly, the upregulation of mRNA translation was not directly regulated by imbalance of tRNA pools. In particular, we showed that alteration in Rho GTPase signaling upon *Mettl1* deletion increased BCAT1 protein level, but how the translation of *Bcat1* mRNA was increased requires further investigation. Consistent with our findings, studies have revealed extensive interaction between BCAT1 and cytoskeleton (31). It has also been reported that BCAA metabolism shapes cytoskeleton and affects Rho GTPase signaling in glial cells (45). Moreover, we found that BCAT1-mediated BCAA degradation promoted oxidative phosphorylation and glycolysis in *Mettl1*-deficient cells. Indeed, a recent study reported reduced oxidative phosphorylation and glycolysis in *Bcat1*-knockdown macrophages (46). A high level of BCAT1 expression was identified in gliomas and leukemia with wild-type IDH, and mimics the mutant IDH phenotype in which aberrant production of 2-hydroxyglutarate competitively inhibits αKG function (47–49). Thus, BCAT1 restricted intracellular αKG levels, which leads to DNA hypermethylation and HIF1α stabilization (47). Whether *Mettl1* deletion enhances glycolysis via the BCAT1/αKG/HIF1α axis remains to be answered. Importantly, we showed that αKG level was restricted in *Mettl1*-deficient cells and its supplementation ameliorated the skeletal defect of *Prrx1^Cre^ Mettl1^fl/fl^* mice, indicating that metabolic alteration by *Mettl1* deletion impairs bone development. αKG performs function via different mechanisms. On one hand, it could extend the lifespan of worms by blocking mitochondrial ATP synthase that inhibits mTOR signaling (50). On the other hand, αKG is a cosubstrate for Fe(II)/αKG-dependent dioxygenases and is capable of modulating the epigenetic landscape (51, 52). For instance, loss of JMJD3, an αKG-dependent H3K27me3 demethylase, severely impairs endochondral ossification by inhibiting chondrocyte proliferation and hypertrophy (53). Therefore, the exact mechanism of the effect of αKG supplementation on bone development of *Prrx1^Cre^ Mettl1^fl/fl^* mice requires further elucidation. Moreover, as *Mettl1* removal affects the abundance of m^7^G-modified tRNAs, which apparently are involved in codon recognition of huge amounts of mRNAs, the bone defect of *Prrx1^Cre^ Mettl1^fl/fl^* mice is unlikely to be caused solely by change in BCAT1 protein level. Therefore, we only observed partial restoration of bone development after αKG supplementation. Other possible mechanisms exist. For example, *Prrx1^Cre^ Mettl1^fl/fl^* mice exhibit brachypodism, which is closely linked to dysregulation of type 1 BMP receptor and growth differentiation factor 5 (GDF5) signaling (54, 55). Though we did not detect direct change in their mRNA translation, whether *Mettl1* deletion affects their downstream signaling remains to be explored.

Besides the selective mRNA translation, we further found that removal of *Mettl1* potently activated ISR and inhibited mTORC1, which reduced translation initiation. ISR is a central hub that relays different stresses to translational control (34, 56). We found that ISR kinase GCN2 was activated in *Mettl1*-deficient cells. GCN2 was initially known as a sensor for amino acid starvation, as it binds to uncharged tRNA, but recent work demonstrates that GCN2 is more potently activated by P-stalk of stalled or colliding ribosomes (57–60). Therefore, we reason that elongation stress evoked by *Mettl1* deletion is sensed by GCN2, which inhibits general translation by phosphorylating eIF2α. This pathological mechanism could be extended to other tRNA-dysregulated conditions that lead to ribosome stalling, such as hypomodification and defect in aminoacyl-tRNA synthetases.

We speculated that manipulating global translation by targeting ISR could correct the defective phenotype. In fact, ISR activation is widely implicated in neurological disorders, including impaired memory formation, Down syndrome, and peripheral neuropathy (36, 61, 62). Inhibition of ISR is sufficient to restore proteostasis and rescue neurological phenotype (36, 62). However, ISR can be protective, as it alleviates the translational stress by reducing ribosome stalling or colliding in circumstances of tRNA deficiency and ribosome defects (58, 63, 64). Currently it is unknown why ISR activation in different contexts reaches opposing outcomes. In this study, we showed that inhibiting ISR by *Gcn2* deletion exacerbated the skeletal defect of *Mettl1*-knockout mice, suggesting a protective role in this circumstance. A recent study demonstrated a ribosome quality control mechanism by which an intermediate level of ribosome colliding activated GCN2-mediated ISR, which supported cell survival, whereas severe colliding activated p38/JNK signaling, which induced apoptosis (65). Based on this model, we propose that ISR is activated for translational quality control upon *Mettl1* deletion. Releasing the brake on translation aggravates ribosome collision and therefore exacerbates the bone defects. The evidence from mTORC1-activated mice supports this model as well. mTORC1 promotes 5′ cap–dependent translation by phosphorylating 4E-BPs (38, 56). Promoting translation by deleting the mTORC1 inhibitor *Tsc1* aggravates the defect of *Prrx1^Cre^ Mettl1^fl/fl^* mice. This finding highlights the context-dependent regulation of ISR and warrants thorough examination of side effects when targeting ISR for therapeutics.

In summary, we demonstrate that METTL1/WDR4-mediated tRNA m^7^G modification is essential to skeletal development. Loss of *Mettl1* downregulates translation of cytoskeletal proteins and impairs Rho GTPase signaling. Impairment of cytoskeleton and Rho GTPase signaling upregulates BCAT1 translation that rewires cell metabolism and restricts αKG. We also reveal inhibition of translation initiation as a protective response to alleviate cellular stress upon tRNA dysregulation. Our study connects tRNA modification to cell metabolism, and suggests that αKG might be a therapeutic option for m^7^G-related primordial dwarfism.

## Methods

Additional methods are provided in Supplemental Methods.

### Sex as a biological variable.

Both male and female animals were examined in this study, and similar findings are reported for both sexes.

### Mouse husbandry and breeding.

CRISPR/Cas9 technique was used to generate *Mettl1^fl/+^* and *Wdr4^R215L/+^* mice on a C57BL6/J background by Beijing Biocytogen Co. Ltd. *Prrx1^Cre^* and *Sp7^Cre^* transgenic mice were purchased from The Jackson Laboratory. *Gcn2^+/–^* (*Eif2ak4^+/–^*) mice were purchased from Gempharmatech Co. Ltd. *Tsc1^fl/+^* mice were a gift from the Hu Zhao laboratory from the Chinese Institute for Brain Research, Beijing, China. For *Prrx1^Cre^-*mediated *Mettl1* knockout, we crossed male *Prrx1^Cre^* mice to female *Mettl1^fl/+^* mice to obtain *Prrx1^Cre^ Mettl1^fl/+^* mice. Male *Prrx1^Cre^ Mettl1^fl/+^* mice were crossed to female *Mettl1^fl/fl^* mice to obtain homozygous *Prrx1^Cre^ Mettl1^fl/fl^* mice. *Mettl1^fl/fl^* mice and *Prrx1^Cre^ Mettl1^fl/+^* mice were used as littermate controls. For embryonic study, mouse vaginal plugs were checked in the morning and counted as E0.5. At planned days, pregnant mice were euthanized with CO_2_, and embryos were then collected for subsequent analyses. For αKG supplementation in drinking water, 1 g αKG was dissolved in 100 mL H_2_O and we adjusted pH (7.4) with NaOH. *Sp7^Cre^* mice were crossed to *Mettl1^fl/+^* mice to obtain *Sp7^Cre^ Mettl1^fl/+^* mice, which were crossed to *Mettl1^fl/fl^* mice to obtain *Sp7^Cre^ Mettl1^fl/fl^* mice. *Sp7^Cre^* mice were used as littermate controls. *Wdr4^R215L/+^* mice were crossed to *Wdr4^R215L/+^* mice to obtain homozygous *Wdr4^R215L/R215L^* mice. WT and *Wdr4^R215L/+^* mice were used as littermate controls. For genotyping, the amplified DNA product was sequenced and checked for mutation from guanine (G) to thymine (T) nucleotide following the AAGATCC sequence. To obtain *Prrx1^Cre^ Mettl1^fl/fl^ Gcn2^–/–^* mice, male *Prrx1^Cre^ Mettl1^fl/+^* mice were crossed to female *Gcn2^–/–^* mice to obtain *Prrx1^Cre^ Mettl1^fl/+^ Gcn2^+/–^* mice. Male *Prrx1^Cre^ Mettl1^fl/+^ Gcn2^+/–^* mice were then crossed to *Mettl1^fl/fl^ Gcn2^–/–^* mice to obtain *Prrx1^Cre^ Mettl1^fl/fl^ Gcn2^–/–^* mice. The same mating strategy was used to obtain *Prrx1^Cre^ Mettl1^fl/fl^ Tsc1^fl/fl^* mice.

### Whole-mount skeletal staining.

The procedure for whole-mount skeletal staining was performed as previously described (66). For E12.5 to E16.5 embryos, the eyeballs were removed, and embryos were fixed in 70% EtOH overnight, then changed to 95% EtOH and to acetone overnight. Embryos were immersed in Alcian blue solution (0.06 g Alcian blue 8GX in 160 mL EtOH and 40 mL acetic acid) for 1–4 hours, and then changed to alizarin red solution (10 mg alizarin red S in 200 mL 1% KOH) for 3–4 hours. Embryos were placed in 1% KOH overnight and then in clearing solution (50:50 of glycerol to 1% KOH). For E16.5 and P0 samples, the skin, eyeballs, internal organs, and adipose tissue were removed. Then the samples were placed in 95% EtOH and then acetone overnight. Embryos were immersed in Alcian blue solution overnight, and were washed twice with 70% EtOH and placed in 95% EtOH overnight. They were placed in 1% KOH for 1 hour and counterstained with alizarin red solution overnight at 4°C. After staining, the samples were subjected to tissue clearing (20:80 of glycerol to 1% KOH) for several days until the skeleton was clearly observed. The samples were transferred to clearing solution (50:50 of glycerol to 1% KOH) for long-term preservation and observed under a stereomicroscope.

### Histology.

Samples were harvested and fixed in 4% paraformaldehyde for 24 hours and then stored in PBS buffer before use. For paraffin slide preparation, samples were decalcified in 10% EDTA (pH 7.4), and were then dehydrated and sectioned (5 μm) as previously described (67). The slides were incubated at 65°C for 1–2 hours, dewaxed with xylene, and rehydrated with gradient EtOH (100%, 95%, 90%, 80%). The slides were subjected to Alcian blue, hematoxylin and eosin, Safranin O, and tartrate-resistant acid phosphatase staining following instructions as previously described (27, 68). For von Kossa staining, embryonic specimens were processed with undecalcified paraffin slides. Postnatal undecalcified specimens were embedded in methylmethacrylate and sectioned (8 μm) with a Leica RM2235 microtome. The slides were then stained with 4% silver nitrate and 0.5% hydroquinone, and counterstained with nuclear fast red.

### Immunohistochemistry and immunofluorescence staining.

Paraffin slides were used for immunohistochemistry. Briefly, after deparaffinization and rehydration, slides were subjected to 3% hydrogen peroxide for 10 minutes and to sodium citrate buffer at 99°C for 15 minutes. The slides were then incubated with blocking buffer for 30 minutes and rabbit anti-METTL1 antibody (1:500) overnight at 4°C. Next day, the slides were incubated with HRP-conjugated goat anti-rabbit secondary antibody and stained with an AEC color development kit (Boster, China). The reaction was stopped and counterstained with hematoxylin.

Frozen sections were used for immunofluorescence. Briefly, the samples were dehydrated with 30% sucrose and embedded with OCT compound for section. The slides were washed with PBS and permeabilized with 0.2% Triton X-100 for 20 minutes. Then they were incubated with blocking buffer for 30 minutes and with corresponding primary antibodies overnight at 4°C, and then with secondary antibodies (Jackson ImmunoResearch). The nuclei were counterstained with DAPI. The slides were mounted and observed under a confocal microscope.

For EdU tracing, EdU powder (Carbosynth) was dissolved in 0.9% NaCl to obtain 1 mg/mL solution, and mice were intraperitoneally injected with EdU solution (10 μL/g) 2 hours before sacrifice. The EdU development cocktail was prepared for staining following the manufacturer’s instruction. A TUNEL assay kit (Promega) was used to detect the apoptotic cells.

### Micro-CT and histomorphometric analyses.

Micro-CT analysis was performed following the guidelines for rodent bone microstructure assessment (69). Femora were placed in a cylindrical sample tube and were scanned by a μCT 80 scanner (Scanco Medical) with a spatial resolution of 8 μm (55 kV, 114 mA, 500 milliseconds integration time). Three-dimensional reconstruction was performed after the scan. For analysis of *Prrx1^Cre^ Mettl1^fl/fl^* mice and control littermates at 3 weeks of age, the regions of interest (ROIs) extended from 200 μm underneath the growth plate to 280 μm (100 slices). For analysis of *Wdr4^R215L/R215L^* mice and control littermates at 6 weeks of age, ROIs extended from 200 mm underneath the growth plate to 320 μm (150 slices). Cortical bone thickness was calculated at the femur diaphysis (25 slices). Bone mineral density (BMD), bone volume fraction (BV/TV), trabecular number (Tb.N), trabecular thickness (Tb.Th), trabecular bone separation (Tb.Sp), and cortical bone thickness (Ct.Th) were calculated within the ROIs. Histomorphometric analyses, including number of osteoblasts per bone perimeter (N.Ob/B.Pm) and number of osteoclasts per bone perimeter (N.Oc/B.Pm), were performed with OsteoMeasure software (OsteoMetrics).

### Cell culture.

For primary culture of embryonic limb mesenchymal progenitors at E14.5, pregnant mice were euthanized, and embryos were carefully dissected and kept in Hanks balanced saline solution. The limbs were then cut from the trunk under a stereomicroscope and predigested with collagenase type II (2 mg/mL) and Dispase (1 mg/mL) dissolved in DMEM/F12 medium by constant agitation for 30 minutes to remove epithelium. A second digestion was then performed for 4 hours, and the suspensions were filtered through a 40 μm mesh. The digested cells were centrifuged, resuspended with DMDM/F12 supplemented with 10% FBS, 100 U/mL penicillin, and 100 μg/mL streptomycin, and cultured in a CO_2_ chamber at 37°C. For primary culture of growth plate chondrocytes at P0, cartilage of the distal femur and proximal tibia was carefully dissected and was predigested for 30 minutes to remove perichondrial cells. The remaining tissue was then digested for 4–5 hours, and the chondrocytes were cultured in α-MEM supplemented with 10% FBS, 100 U/mL penicillin, and 100 μg/mL streptomycin. For primary culture of bone marrow SSCs, the femur and tibia were dissected, and bone marrow tissue was flushed out using a syringe and cultured with α-MEM supplemented with 15% FBS, 100 U/mL penicillin, and 100 μg/mL streptomycin. SSCs were passaged 3 times before osteogenic induction.

### Cell immunofluorescence.

Cells cultured in coverslips were washed with PBS and fixed with 4% paraformaldehyde for 20 minutes, and then were permeabilized with 0.2% Triton X-100 for 10 minutes. Then they were incubated with 5% BSA blocking buffer for 30 minutes and with corresponding primary antibodies overnight at 4°C, and then with secondary antibodies (Jackson ImmunoResearch). The cytoskeleton was stained with phalloidin, and the nuclei were counterstained with DAPI. The slides were mounted and observed under a confocal microscope.

### ALP and alizarin red S staining.

For osteogenic induction, SSCs were treated with osteogenic medium containing 5 mM β-glycerophosphate, 50 μg/mL ascorbic acid, and 100 nM dexamethasone. ALP staining was performed using a BCIP/NBT staining kit, and ALP activity was measured with an ALP assay kit (Beyotime) according to the manufacturer’s instructions after osteogenic induction for 10 days. Alizarin red S staining and quantification were performed after osteogenic induction for 14 days. Briefly, cells were fixed with 4% paraformaldehyde for 15 minutes and stained with 1% alizarin red S solution for 5–15 minutes. The calcified nodules were then destained by 10% cetylpyridinium chloride solution, and the calcium concentration was measured by a microplate reader at 562 nm.

### Western blot, Northwestern blot, and Northern blot.

For Western blot, tissue and cells were lysed with RIPA lysis buffer supplemented with inhibitors of protease and phosphatase. A bicinchoninic acid assay kit was used to determine the protein centration. Samples were separated by SDS-PAGE polyacrylamide gels and electrotransferred to PVDF membrane. The membrane was then blocked with 8% nonfat milk for 1 hour and incubated with primary antibodies overnight at 4°C. The next day, membrane was incubated with HRP-conjugated secondary antibodies (1:5,000) for 1 hour and finally visualized with ECL reagents using ChemiDoc Imaging Systems (Bio-Rad). For Northern blot, RNAs were extracted using TRIzol reagent and separated by tris-borate-EDTA-urea gels (Bio-Rad). The separated RNAs were then transferred to positively charged nylon membrane and subjected to UV light for cross-linking. The membrane was then incubated with digoxigenin-labeled probes against U6 small nuclear RNA and tRNAs. The RNA probes are listed in Supplemental Table 2. For Northwestern blot, the membrane was blotted with anti-m^7^G antibody (1:2,000; MBL International), and then the m^7^G signal was detected following the Western blot procedure.

### Protein synthesis assay.

The cells were incubated with 1 μM puromycin for 20 minutes. Anti-puromycin antibody was used to detect the protein synthesis rate by Western blot (70).

### Quantitative reverse transcriptase PCR.

Total RNA was extracted using TRIzol reagent according to the manufacturer’s instructions. RNA concentration and quality were assessed with a NanoDrop 2000 spectrophotometer (Thermo Fisher Scientific). RNA was reverse-transcribed to cDNA using PrimeScript RT Reagent Kit with gDNA Eraser (Takara). Quantitative PCR was then performed using SYBR Premix Ex Taq II (Takara) in a CFX96 Real-Time System (Bio-Rad). The 2^−ΔΔCt^ method was used to calculate relative gene expression, and *Gapdh* was used for normalization. Primers for quantitative reverse transcriptase PCR (qRT-PCR) are listed in Supplemental Table 3.

### RNA sequencing.

Total RNA was extracted by TRIzol reagent. mRNA was purified from the total RNA, and the NEBNext Ultra RNA Library Prep Kit for Illumina (New England Biolabs) was used for library construction, followed by sequencing on the Illumina HiSeq 2500 platform. FastQC (https://www.bioinformatics.babraham.ac.uk/projects/fastqc/) and FASTX (https://github.com/agordon/fastx_toolkit) were used for quality control, and the sequencing data were mapped to *Mus musculus* reference genome using HISAT2 (https://daehwankimlab.github.io/hisat2/). DESeq2 (https://bioconductor.org/packages/release/bioc/html/DESeq2.html) was used for analysis of differentially expressed genes. Gene expression was considered differentially expressed when fold change was greater than 1 or less than –1 and adjusted *P* value was less than 0.05. *Cis*-regulatory analysis was conducted with iRegulon (http://iregulon.aertslab.org/). Gene set enrichment analysis (GSEA) was performed using GSEA software (UCSD and Broad Institute).

### tRNA reduction and cleavage sequencing.

We followed the protocol for m^7^G tRNA reduction and cleavage sequencing (TRAC-Seq) (29). Briefly, total RNA was extracted by TRIzol reagent, and small RNA was purified with a Quick-RNA Microprep Kit (Zymo Research). The m^1^A, m^3^C, and m^1^G modifications were removed using AlkB-WT and AlkB-D135S recombinant proteins. The m^7^G site was reduced with 0.2 M sodium borohydride (NaBH_4_) and cut with aniline lysates. NEBNext Small RNA Library Prep Set kit for Illumina (New England Biolabs) was used to construct a cDNA library, which was subjected to sequencing. FastQC and Trim Galore (https://github.com/FelixKrueger/TrimGalore) were used for quality control. Bowtie (https://bowtie-bio.sourceforge.net/index.shtml) was used for human tRNA genome mapping. Bedtools (https://bedtools.readthedocs.io/en/latest/) calculated the sequencing depth of each tRNA site. Cleavage score and cleavage ratio were calculated by cleavage_score.R. An m^7^G site was identified when the cleavage score was greater than 6 and the cleavage ratio was greater than 0.1. MEME (Multiple Em for Motif Elicitation) was used for m^7^G motif analysis. DESeq2 was used to calculate tRNA expression change.

### Ribosome profiling.

Cells were treated with 100 μg/mL cycloheximide, lysed, and centrifuged to collect the supernatant, which was then nuclease-digested with RNase I and DNase I to obtain the ribosome-protected fragments (RPFs). RPFs were separated by the RNA Clean and Concentrator-25 kit and purified by magnetic beads. The library was constructed using NEBNext Multiplex Small RNA Library Prep Set for Illumina (New England Biolabs). The ribosome profiling data were analyzed with RiboToolkit (http://rnainformatics.org.cn/RiboToolkit/) (71).

### Adenovirus infection.

The adenoviruses encoding mouse *Cre* or green fluorescent protein (*gfp*) were purchased from Genechem (China). Bone marrow SSCs were cultured on 6-well plate at a density of 2 × 10^4^ cells/cm^2^. When 60% confluence was reached, SSCs were infected with Ad-*Cre* or Ad-*gfp* at multiplicity of infection of 30 for 12 hours. SSCs were changed into α-MEM complete medium.

### OCR and ECAR metabolic assays.

Oxygen consumption rate (OCR) and extracellular acidification rate (ECAR) were measured on a Seahorse XF24 analyzer (Agilent). The assay medium for OCR was unbuffered DMEM (pH 7.4, with or without BCAA) supplemented with 5 mM d-glucose, 1 mM sodium pyruvate, and 2 mM l-glutamine. The assay medium for ECAR was unbuffered DMEM (pH 7.4) supplemented with 2 mM l-glutamine. The Seahorse XF Cell Mito Stress Test Kit and Seahorse XF Glycolytic Rate Assay Kit were used for OCR and ECAR assays.

### Measurements of ATP, glucose, and lactate.

Intracellular ATP was measured following the instructions of the ATP assay kit (Beyotime, China). For glucose consumption and lactate production test, cells were cultured in basal DMEM (glucose free, pyruvate free, Gibco) supplemented with 5 mM glucose, 1 mM sodium pyruvate, 10% dialyzed FBS, 100 U/mL penicillin, and 100 μg/mL streptomycin. After 24-hour culture, the culture medium was collected and tested with a glucose assay kit (Nanjing Jiancheng Bioengineering Institute) and a lactate assay kit (Solarbio). The results were normalized to protein amount.

### Untargeted metabolomics.

Untargeted metabolomics was performed by Applied Protein Technology. Briefly, limb mesenchymal stem progenitors cultured on a dish were scraped off on ice, and the metabolites were extracted with cold methanol/acetonitrile (1:1, vol/vol). After centrifugation, the supernatant was lyophilized. For liquid chromatography–mass spectrometry (LC–MS) analysis, samples were dissolved in acetonitrile/H_2_O (1:1, vol/vol), separated by ultra-high-performance liquid chromatography (1290 Infinity LC, Agilent), and coupled to TripleTOF 6600 quadrupole time-of-flight mass spectrometry (SCIEX). The data were converted to mzXML files and processed with XCMS software (Mass Consortium Corporation). The data were then subjected to orthogonal partial least-squares discriminant analysis (OPLS-DA), and the variable importance in the projection (VIP) value of each metabolite in the OPLS-DA model was calculated. Student’s 2-tailed *t* test was used to calculate the significance of difference between WT and *Prrx1^Cre^ Mettl1^fl/fl^* groups. The metabolite level was considered significantly changed when VIP was greater than 1 and *P* value was less than 0.05.

### Metabolic flux analysis of [U-^13^C]glucose and [^13^C,^15^N]l-leucine.

The metabolic flux analysis was performed by Applied Protein Technology. Briefly, for [U-^13^C]glucose analysis, limb mesenchymal progenitors at a density of 70%–80% were incubated with basal DMEM supplemented with 5 mM stable isotope–labeled [U-^13^C]glucose, 1 mM sodium pyruvate, and 10% dialyzed FBS. After 60 minutes of culture, the supernatant was discarded, and cells were rinsed 3 times with cold PBS. The extraction solution (methanol/acetonitrile, 1:1, vol/vol) was added, and cells were scraped, sonicated, and centrifuged to obtain the supernatant, which was lyophilized. For [^13^C,^15^N]leucine analysis, limb mesenchymal progenitors at a density of 70%–80% were incubated with leucine-free DMEM supplemented with 0.8 mM stable isotope–labeled [^13^C,^15^N]l-leucine, 5 mM glucose, 1 mM sodium pyruvate, and 10% dialyzed FBS. For LC-MS/MS analysis, samples were dissolved in methanol/H_2_O (1:1, vol/vol) and analyzed by a Vanquish UPLC system (Thermo Fisher Scientific) coupled to an Exactive Plus Hybrid Quadrupole-Orbitrap Mass Spectrometer (Thermo Fisher Scientific). Data were analyzed by TraceFinder 5.0 (Thermo Fisher Scientific) and Xcalibur 4.0 (Thermo Fisher Scientific). The metabolic mass distribution vector of a given metabolite was calculated by division of the integrated peak areas (IPAs) of the selected isotopomer by the sum of all IPAs of possible isotopomers and was then corrected for natural abundance.

### Mitochondrial mass and ROS analysis.

For evaluation of mitochondrial mass and mitochondrial ROS production, cells were incubated with 100 nM MitoTracker Red CMXRos (Yeasen) for 30 minutes at 37°C or 5 μM MitoSOX Red mitochondrial superoxide indicator (Invitrogen) for 10 minutes at 37°C. After trypsinization, cells were subjected to flow cytometry analysis (Attune NxT, Thermo Fisher Scientific).

### Statistics.

All data are expressed as mean ± SEM. For comparison between 2 groups, unpaired 2-tailed Student’s *t* test was performed. For multiple comparison, 1-way ANOVA followed by Tukey’s post hoc test or 2-way ANOVA followed by Bonferroni’s post hoc test was performed. *P* values less than 0.05 were considered statistically significant.

### Study approval.

Animal experiments were performed in compliance with protocols approved by the Subcommittee on Research and Animal Care of Sichuan University. All mice were housed under specific pathogen–free conditions with ad libitum access to food and drinking water. Mouse tails were collected to extract DNA for genotyping. Primers are listed in Supplemental Table 1.

### Data availability.

The data that support the findings of this study are available within the article and its supplemental material. The raw sequencing data of m^7^G TRAC-Seq, ribosome profiling, and untargeted metabolomics were deposited in the Genome Sequence Archive in the National Genomics Data Center under accession numbers CRA012901, CRA012883, and OMIX005049. Values for all data points in graphs are reported in the Supporting Data Values file. Any additional information required to reanalyze the data reported in this paper is available upon request.

## Author contributions

QL, SL, DC, and QY designed the experiments. QL and SJ performed most of the experiments and interpreted the data. KL was involved in animal breeding, sample collection, and data interpretation. HH participated in m^7^G TRAC-Seq, Northern blot, and Northwestern blot. YC, WL, and QX participated in in vivo experiments and bioinformatics analysis. XQ and RS were involved in primary cell culture, qRT-PCR, and Western blot. XG and YW participated in metabolic and αKG rescue experiments. YZ and TL conducted Seahorse metabolic assays. JM was involved in m^7^G TRAC-Seq. CZ, DC, and QY supervised the project. QL, SL, DC, and QY drafted and revised the manuscript.

## Supplementary Material

Supplemental data

Supplemental table 4

Supplemental video 1

Supporting data values

## Version 1 09/10/2024

In-Press Preview

## Version 2 10/15/2024

Electronic publication
