# Supplementary material for: Metabolic rewiring during bone development underlies tRNA m7G–associated primordial dwarfism
Source: J Clin Invest. 2024 Sep 10;134(20):e177220. doi: 10.1172/JCI177220 (PMC11473147; doi:10.1172/JCI177220)
Supplement: Supplemental data [file jci-134-177220-s148.pdf]

Supplemental Information

Metabolic rewiring during bone development underlies tRNA m^{7}G-associated primordial dwarfism

Qiwen Li[1,5], Shuang Jiang[1,5], Kexin Lei[1], Hui Han[2], Yaqian Chen[1], Weimin Lin[1], Qiuchan Xiong[1], Xingying Qi[1], Xinyan Gan[1], Rui Sheng[1], Yuan Wang[1], Yarong Zhang[1], Jieyi Ma[2], Tao Li[3], Shuibin Lin[2], Chenchen Zhou[1,*], Demeng Chen[4,*] and Quan Yuan[1,*]

[1]State Key Laboratory of Oral Diseases & National Center for Stomatology & National Clinical Research Center for Oral Diseases, West China Hospital of Stomatology, Sichuan University

[2]Center for Translational Medicine, Precision Medicine Institute, The First Affiliated Hospital, Sun Yat-sen University, Guangzhou 510080, China.

[3]West China-Washington Mitochondria and Metabolism Center and Department of Anesthesiology, West China Hospital, Sichuan University, Chengdu 610041, China.

[4]Otorhinolaryngology Hospital, The First Affiliated Hospital, Sun Yat-sen University, Guangzhou 510080, China.

[5]These authors contributed equally.

*Correspondence to Quan Yuan (lead contact, yuanquan@scu.edu.cn), Demeng Chen (chendm29@mail.sysu.edu.cn) and Chenchen Zhou (chenchenzhou5510@scu.edu.cn).

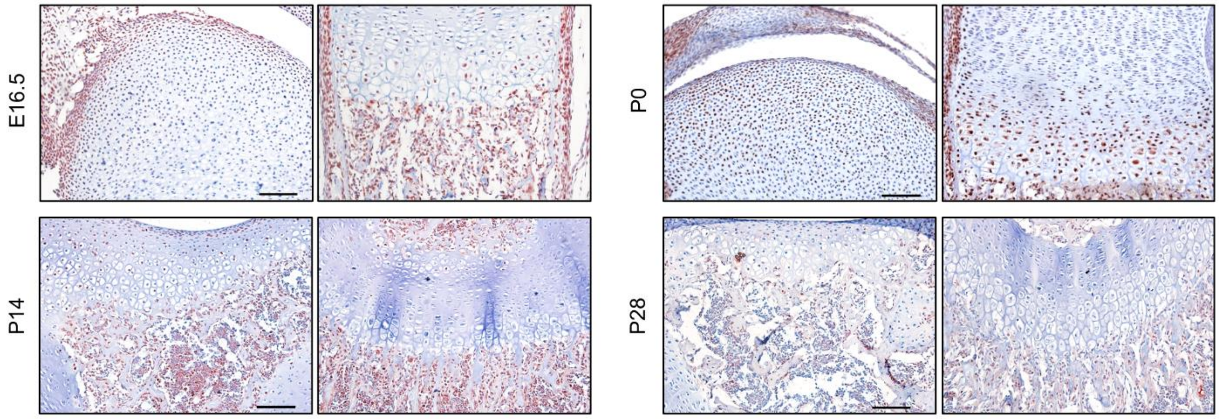

Figure S1 METTL1 expression in mouse long bone

Representative immunohistological staining of METTL1 expression in femur at different time points. Scale bar, 50 μm.

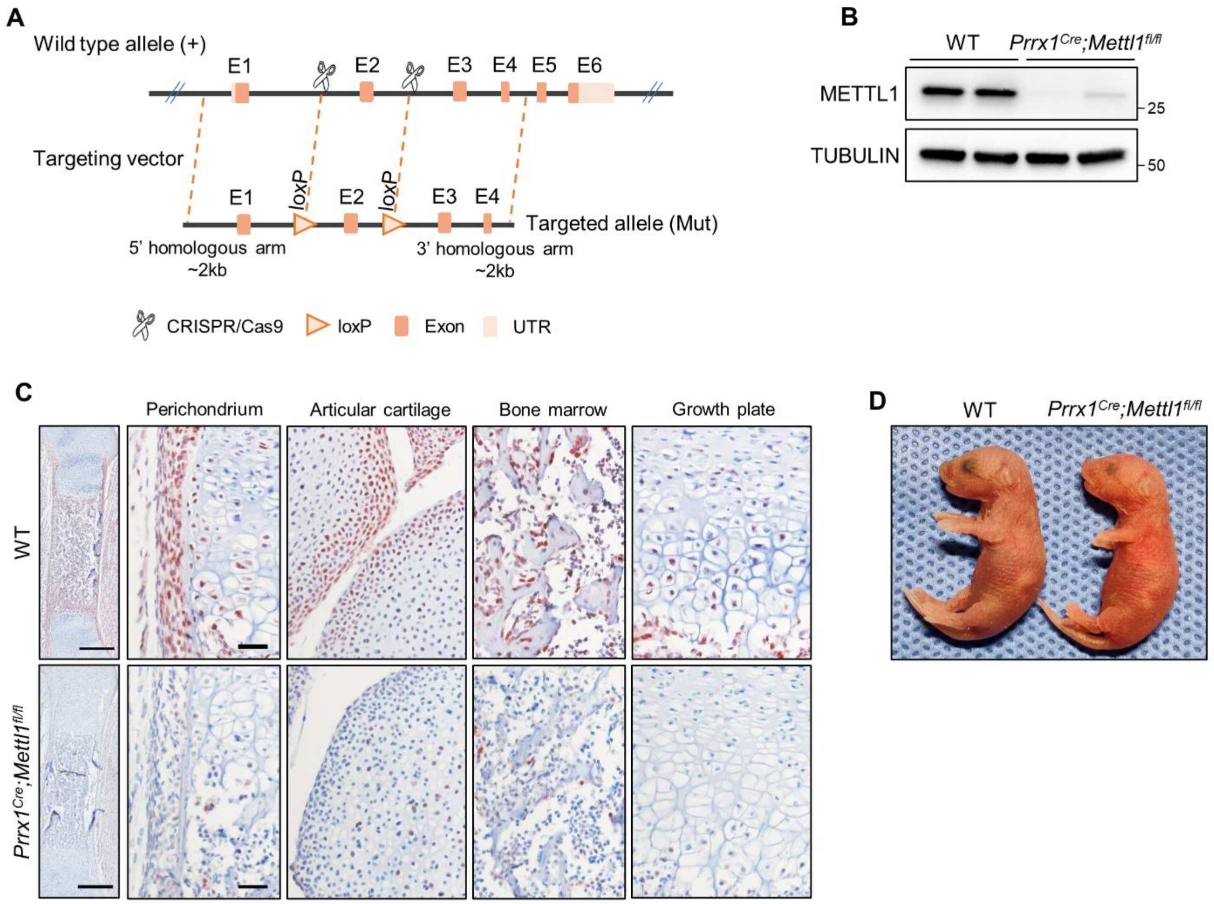

Figure S2 Construction of *Prrx1*^{Cre}*;Mettl1*$^{fl/fl}$ mice

(A) Schematic representation of *Mettl1* conditional knockout strategy. Exon 2 is deleted upon Cre-mediated recombination.

(B) Western blot analysis of METTL1 expression in WT and *Prrx1*^{Cre}*;Mettl1*$^{fl/fl}$ mouse limb tissue lysates at E14.5.

(C) Representative immunohistological staining of METTL1 in WT and *Prrx1*^{Cre}*;Mettl1*$^{fl/fl}$ mouse femur at P0. Scale bar, 200 µm (left) and 20 µm (right).

(D) Representative images of WT and *Prrx1*^{Cre}*;Mettl1*$^{fl/fl}$ mice at P0.

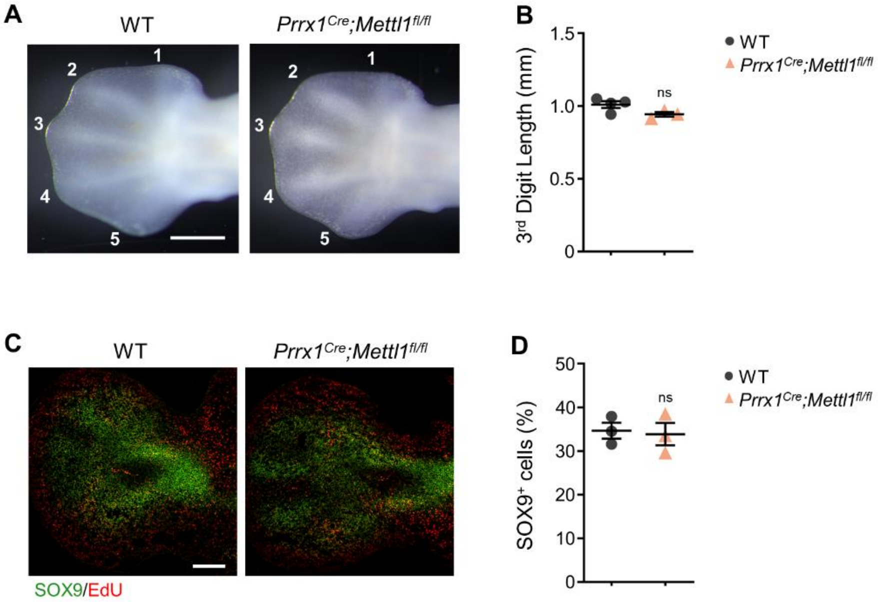

Figure S3 Deletion of *Mettl1* does not impair limb patterning

(A) Representative images of WT and *Prrx1^{Cre};Mettl1$^{fl/fl}$* mouse forelimb bud at E12.5. Scale bar, 500 µm.

(B) Quantification of third digit length of WT and *Prrx1^{Cre};Mettl1$^{fl/fl}$* mouse forelimb bud at E12.5. n = 4 (WT) or 3 (*Prrx1^{Cre};Mettl1$^{fl/fl}$*).

(C & D) Representative immunostaining and quantification of SOX9 in WT and *Prrx1^{Cre};Mettl1$^{fl/fl}$* mouse forelimb bud at E12.5. Scale bar, 200 µm. n = 3.

Data are expressed as mean ± s.e.m; *P* value was calculated by two-tailed Student's *t* test. ns, not significant.

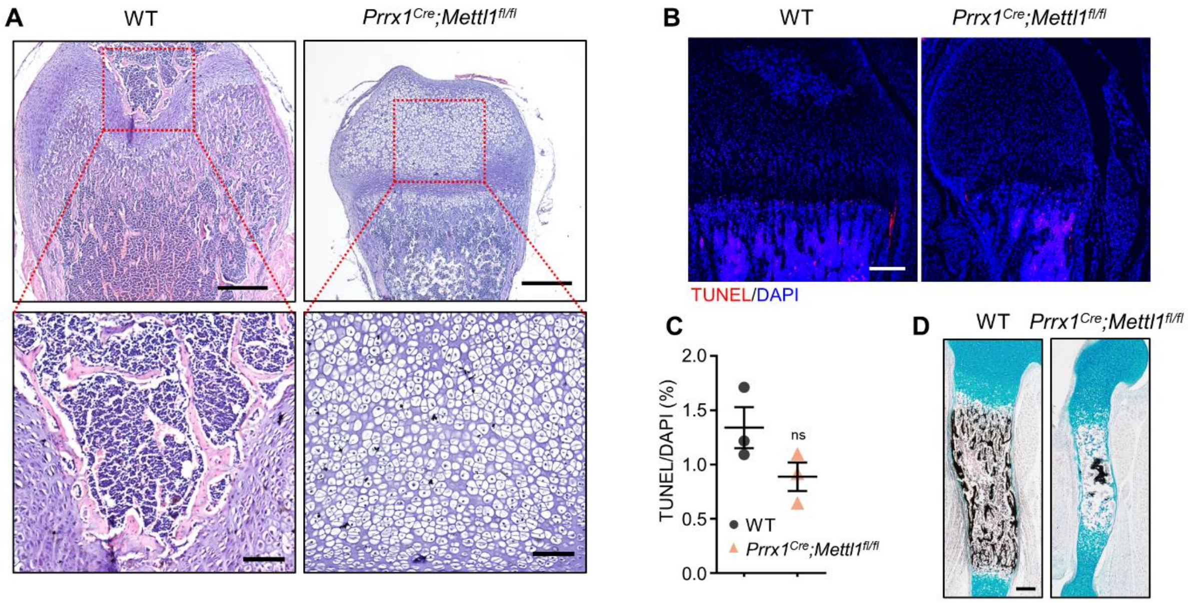

Figure S4 Deletion of *Mettl1* inhibits bone formation

(A) H&E staining of WT and *Prrx1*^{Cre}*;Mettl1*$^{fl/fl}$ mouse femur at P21. Scale bar, 200 μm (upper) and 50 μm (lower).

(B & C) Terminal deoxynucleotidyl transferase dUTP nick end labeling (TUNEL) assay and quantification of WT and *Prrx1*^{Cre}*;Mettl1*$^{fl/fl}$ mouse humerus at P9. Scale bar, 200 μm. n = 3.

(D) Representative Alcian blue/von kossa staining of WT and *Prrx1*^{Cre}*;Mettl1*$^{fl/fl}$ mouse humerus at E18.5. Scale bar, 100 μm.

Data are expressed as mean ± s.e.m; *P* value was calculated by two-tailed Student's *t* test. ns, not significant.

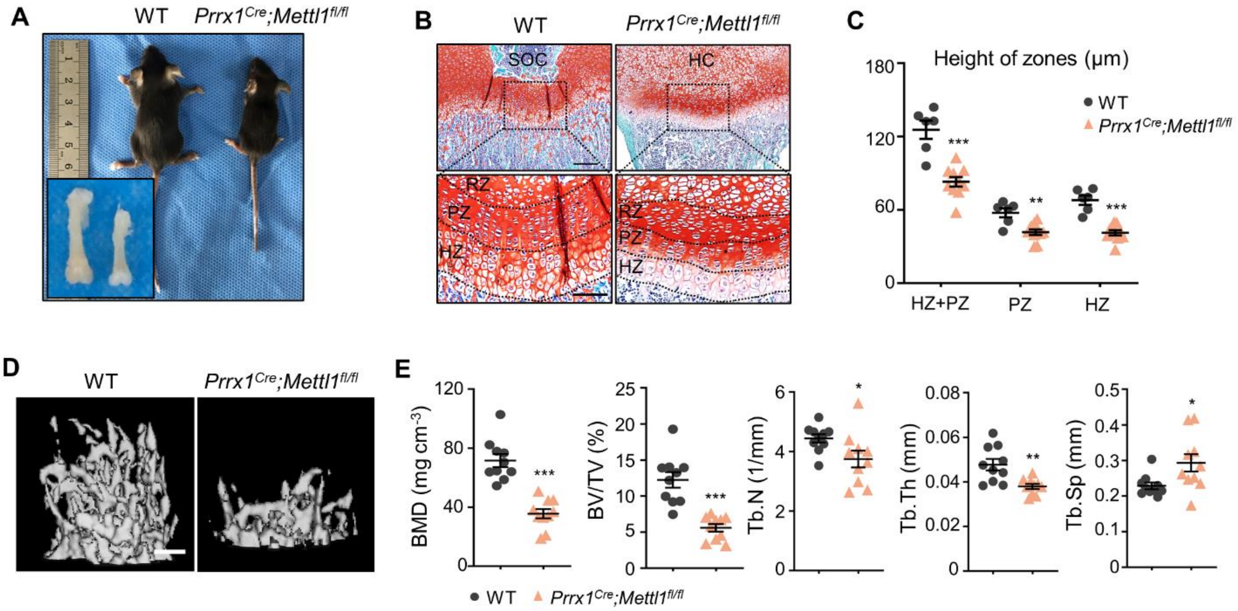

Figure S5 Deletion of *Mettl1* inhibits bone growth and reduces bone mass

(A) Representative images of WT and *Prrx1*^{Cre}*;Mettl1*$^{fl/fl}$ mice at 3 weeks of age. Inserted image was femur of WT and *Prrx1*^{Cre}*;Mettl1*$^{fl/fl}$ mice.

(B & C) Representative Safranin O staining and quantification of WT and *Prrx1*^{Cre}*;Mettl1*$^{fl/fl}$ mouse growth plate of the distal femur at 3 weeks of age. Boxed areas were magnified. SOC, secondary ossification center; RZ, resting zone; PZ, proliferating zone; HZ, hypertrophic zone. Scale bar, 100 μm (upper) and 50 μm (lower). n = 6 for WT and n = 10 for *Prx*^{Cre}*;Mettl1*$^{fl/fl}$.

(D & E) Representative μCT images and quantification of femurs from WT and *Prrx1*^{Cre}*;Mettl1*$^{fl/fl}$ mice at 3 weeks of age. Scale bar, 200 μm. n = 10.

Data are expressed as mean ± s.e.m; *$P < 0.05$, **$P < 0.01$, ***$P < 0.001$ by two-tailed Student's *t* test.

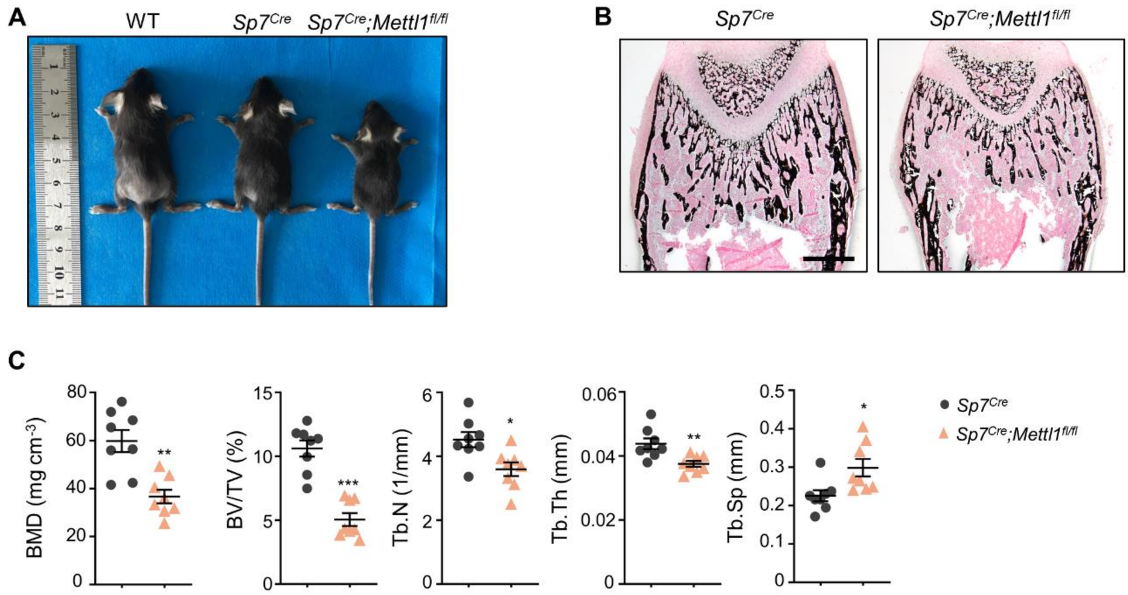

Figure S6 Deletion of *Mettl1* in osteoblast precursors inhibits bone growth

(A) Representative images of WT, *SP7*^{Cre} and *Sp7*^{Cre}*;Mettl1*$^{fl/fl}$ mice at 3 weeks of age.

(B) Representative von kossa staining of femurs from *Sp7*^{Cre} and *Sp7*^{Cre}*;Mettl1*$^{fl/fl}$ mice at 3 weeks of age. Scale bar, 200 μm.

(C) Representative μCT quantification of femurs from *Sp7*^{Cre} and *Sp7*^{Cre}*;Mettl1*$^{fl/fl}$ mice at 3 weeks of age. n = 8.

Data are expressed as mean ± s.e.m; **P* < 0.05, ***P* < 0.01, ****P* < 0.001 by two-tailed Student's *t* test.

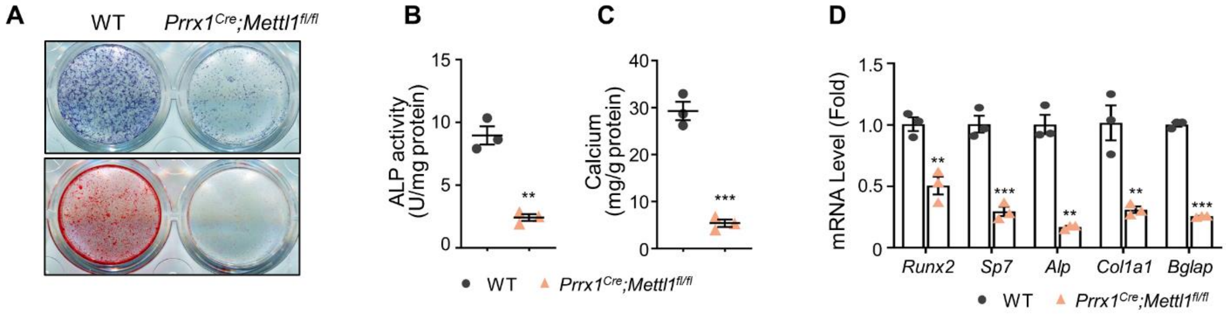

Figure S7 Deletion of *Mettl1* inhibits osteogenesis

(A-C) Representative images and quantification of alkaline phosphatase (ALP) and alizarin red S (ARS) staining of bone marrow SSCs isolated from WT and *Prrx1*^{Cre};*Mettl1*$^{fl/fl}$ mice at 3 weeks of age. n = 3 from independent experiments.

(D) qRT-PCR analyses of the expression of *Runx2*, *Sp7*, *Alp*, *Col1a1* and *Bglap* after osteogenic induction of WT and *Prrx1*^{Cre};*Mettl1*$^{fl/fl}$ SSCs for 5 days. n = 3 from independent experiments.

Data are expressed as mean ± s.e.m; *P < 0.05, **P < 0.01, ***P < 0.001 by two-tailed Student's t test.

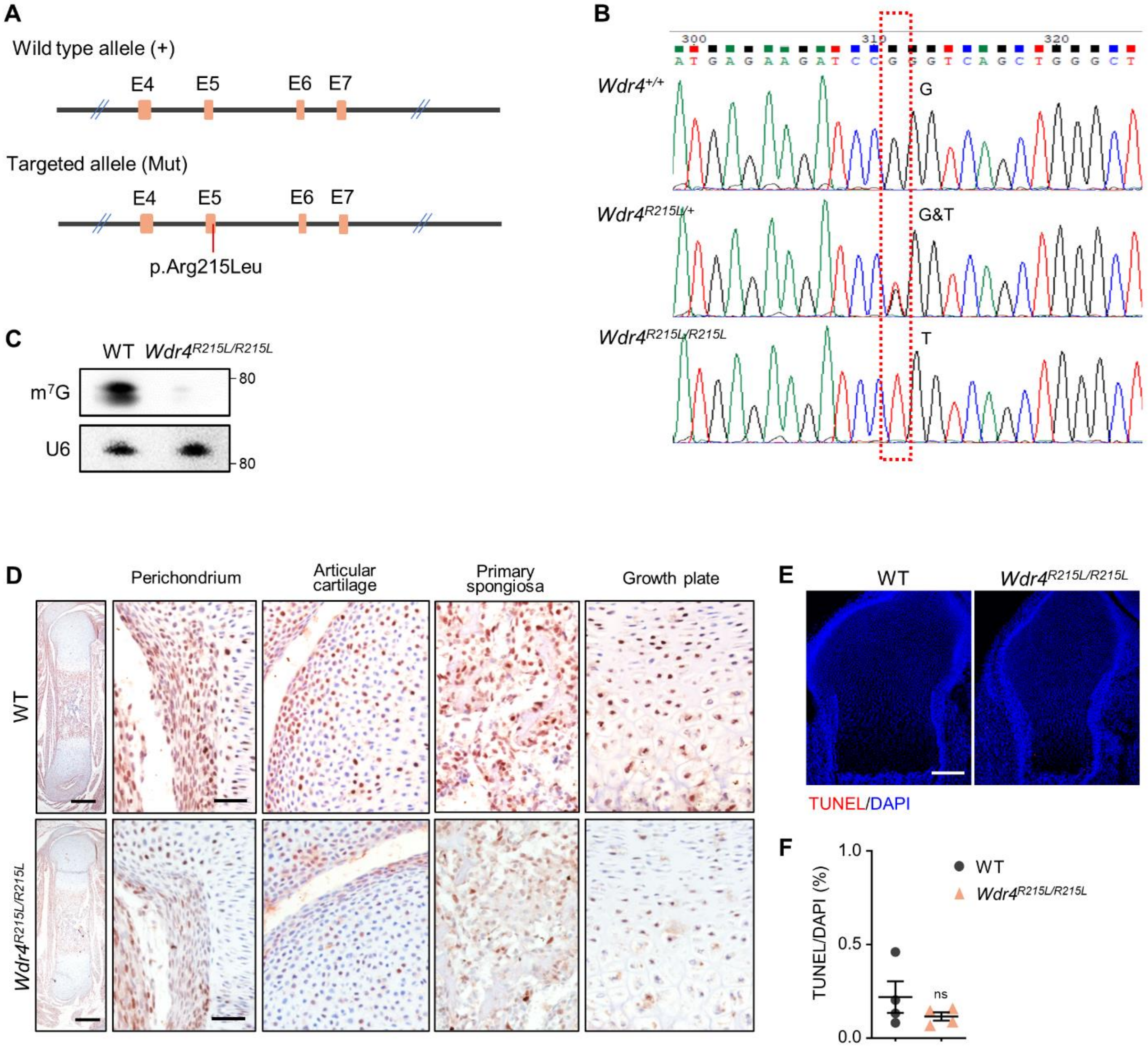

Figure S8 Construction of *Wdr4*$^{R215L/R215L}$ mice

(A) Schematic representation of *Wdr4* mutation.

(B) Representative DNA sequencing results of WT, *Wdr4*$^{R215L/+}$ and *Wdr4*$^{R215L/R215L}$ mice. Boxed areas were mutation site.

(C) m^7G northwestern blot of WT and *Wdr4*$^{R215L/+}$ mouse limb mesenchymal progenitors. U6 snRNA was used as loading control.

(D) Representative immunohistological staining of METTL1 in WT and *Wdr4*$^{R215L/R215L}$ mouse femur at P0. Scale bar, 200 μm (left) and 20 μm (right).

(E & F) TUNEL assay and quantification of WT and *Wdr4*$^{R215L/R215L}$ mouse femur at E18.5. Scale bar, 200 μm.

Data are expressed as mean ± s.e.m; *P* value was calculated by two-tailed Student's *t* test. ns, not significant.

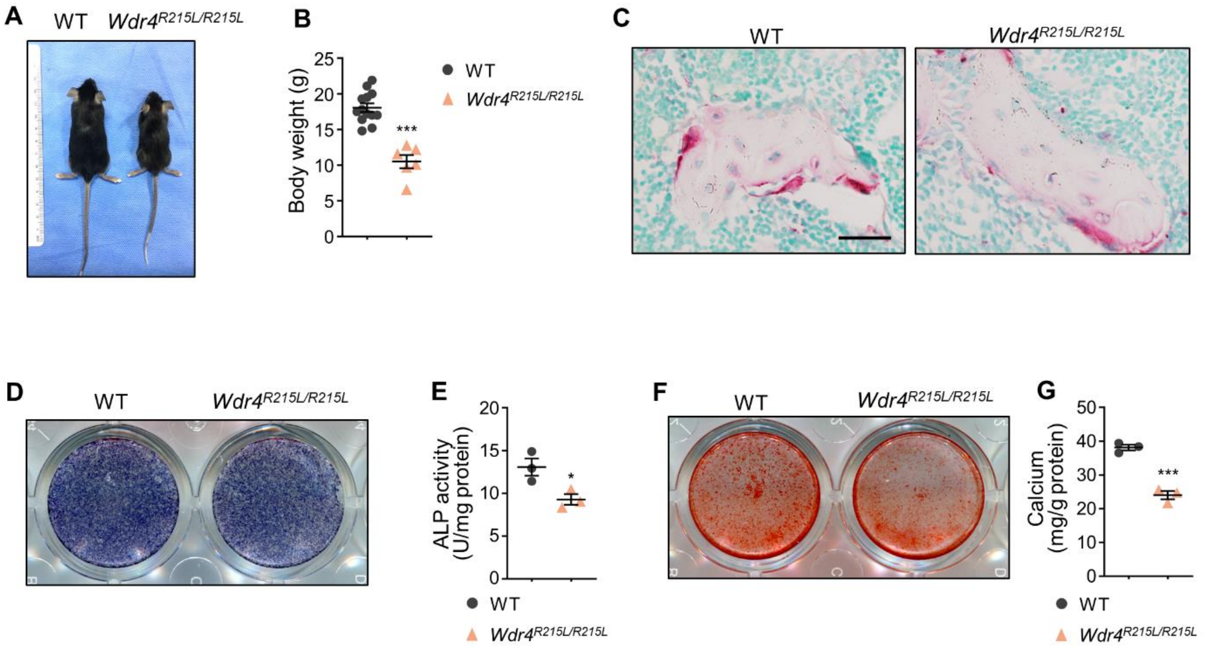

Figure S9 Homozygous *Wdr4*^{R215L} mutation inhibits osteogenesis

(A & B) Representative image and body weight of WT and *Wdr4*$^{R215L/R215L}$ mice at 6 weeks of age. n = 13 for WT and n = 6 for *Wdr4*$^{R215L/R215L}$ mice.

(C) Representative TRAP staining of WT and *Wdr4*$^{R215L/R215L}$ mouse femur at 6 weeks of age. Scale bar, 20 μm.

(D-G) Representative images and quantification of ALP and ARS staining of bone marrow SSCs isolated from WT and *Wdr4*$^{R215L/R215L}$ mice at 6 weeks of age. n = 3 from independent experiments. Data are expressed as mean ± s.e.m; *$P < 0.05$, **$P < 0.01$, ***$P < 0.001$ by two-tailed Student's *t* test.

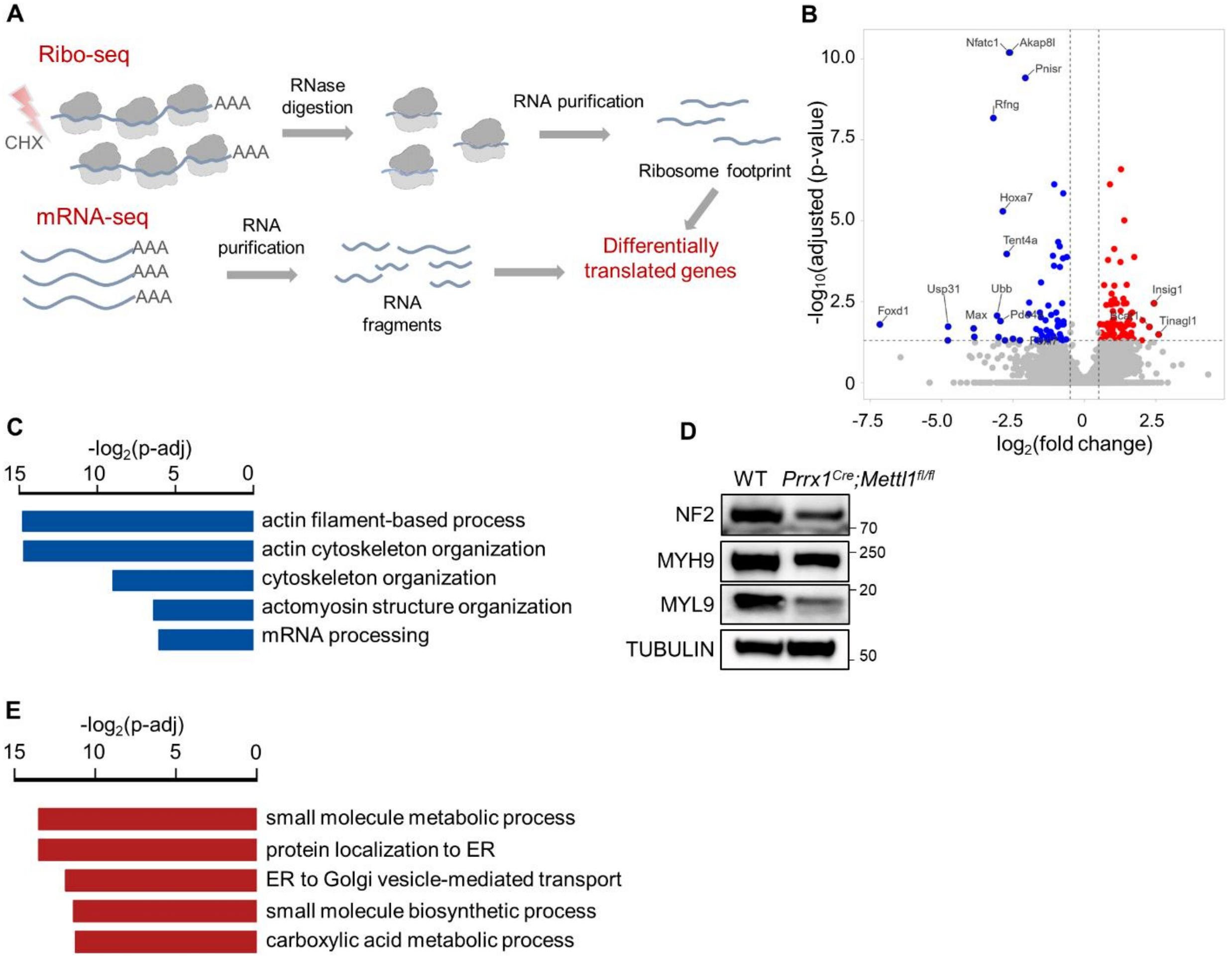

Figure S10 Ribosome profiling of *Mettl1*-deficient cells

(A) Schematic representation of ribosome profiling.

(B) Volcano plot of differentially translated genes in *Prrx1*^{Cre};*Mettl1*$^{fl/fl}$ limb mesenchymal progenitors at E14.5.

(C) Gene ontology (GO) analysis of down-translated genes in *Prrx1*^{Cre};*Mettl1*$^{fl/fl}$ limb mesenchymal progenitors.

(D) Western blot analysis of cytoskeleton proteins in WT and *Prrx1*^{Cre};*Mettl1*$^{fl/fl}$ chondrocytes.

(E) GO analysis of up-translated genes in *Prrx1*^{Cre};*Mettl1*$^{fl/fl}$ limb mesenchymal progenitors.

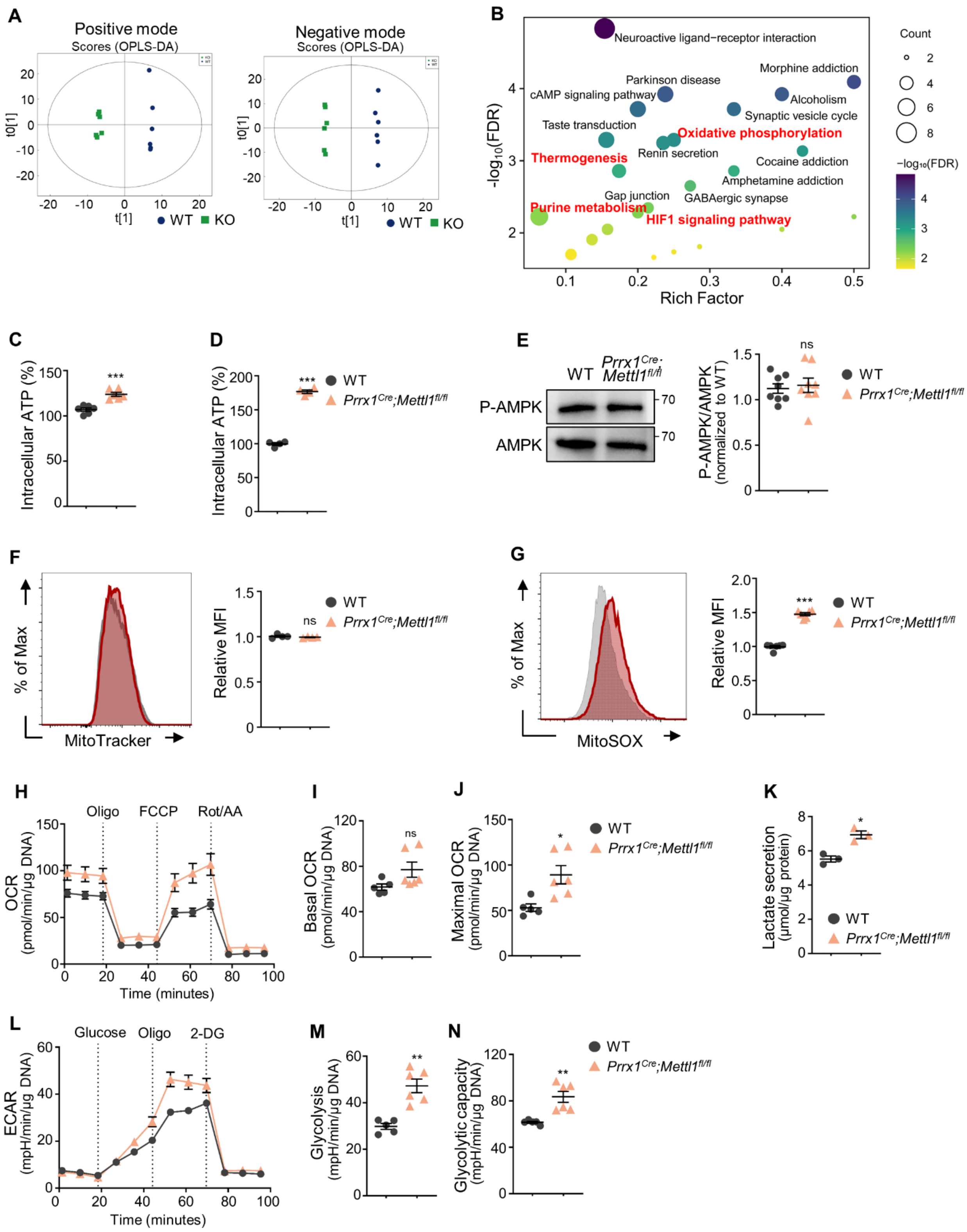

Figure S11 *Mettl1* deletion promotes cellular respiration

(A) OPLS-DA analysis of WT and *Prrx1*^{Cre};*Mettl1*$^{fl/fl}$ group.

(B) Pathway enrichment analysis of differential metabolites upon *Mettl1* removal.

(C) Quantification of intracellular ATP levels in WT and *Prrx1*^{Cre}*;Mettl1*$^{fl/fl}$ limb mesenchymal progenitors at E14.5. n = 6.
(D) Quantification of intracellular ATP levels in WT and *Prrx1*^{Cre}*;Mettl1*$^{fl/fl}$ chondrocytes at P0. n = 4.
(E) Western blot analysis and quantification of phospho-AMPK and AMPK levels. n = 8.
(F) Flow cytometry analysis of WT and *Prrx1*^{Cre}*;Mettl1*$^{fl/fl}$ cells stained with MitoTracker. n = 4.
(G) Flow cytometry analysis of WT and *Prrx1*^{Cre}*;Mettl1*$^{fl/fl}$ cells stained with MitoSOX. n = 6.
(H-J) Representative graph of oxygen consumption rate (OCR) and quantification at baseline and with indicated drugs. Oligo, oligomycin; FCCP, Carbonyl cyanide-4 (trifluoromethoxy) Phenylhydrazone; Rot/AA, rotenone and antimycin A. n = 5 (WT) or 6 (*Prrx1*^{Cre}*;Mettl1*$^{fl/fl}$).
(K) Quantification of lactate secretion in WT and *Prrx1*^{Cre}*;Mettl1*$^{fl/fl}$ limb mesenchymal progenitors.
(L-N) Representative graph of extracellular acidification rate (ECAR) and quantification at baseline and with indicated drugs. 2-DG, 2-deoxy-glucose. n = 5 (WT) or 6 (*Prrx1*^{Cre}*;Mettl1*$^{fl/fl}$).
Data are expressed as mean ± s.e.m; **P* < 0.05, ***P* < 0.01, ****P* < 0.001 by two-tailed Student's *t* test. ns, not significant.

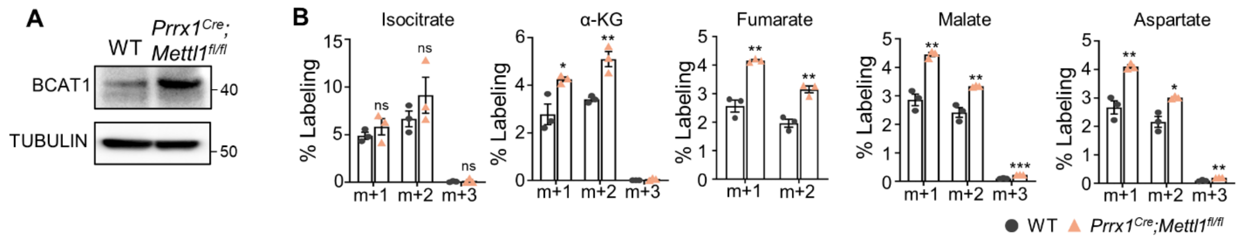

Figure S12 *Mettl1* deletion promotes BCAA catabolism

(A) Western blot analysis of BCAT1 levels in WT and *Prrx1^{Cre};Mettl1$^{fl/fl}$* limb mesenchymal progenitors.

(B) Abundance of ^{13}C-labeled intermediate metabolites in TCA cycle of cells treated with ^{13}C,^{15}N-L-Leucine. n = 3. Data are expressed as mean ± s.e.m; **P* < 0.05, ***P* < 0.01, ****P* < 0.001 by two-tailed Student's *t* test. ns, not significant.

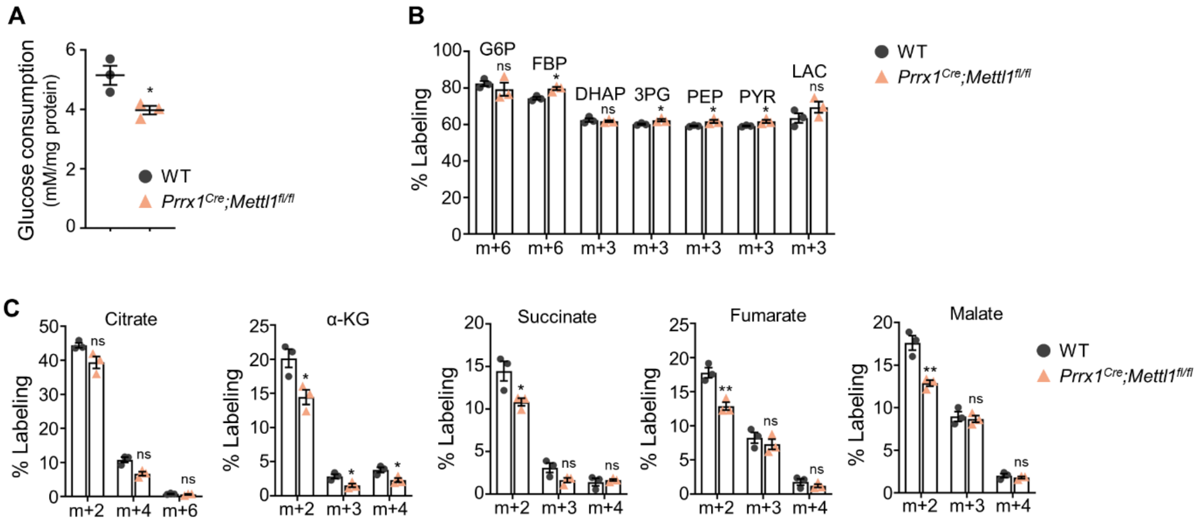

Figure S13 *Mettl1* deletion inhibits glucose metabolism

(A) Measurement of glucose consumption in WT and *Prrx1*^{Cre};*Mettl1*$^{fl/fl}$ limb mesenchymal progenitors at E14.5. n = 3 from independent experiments.

(B) Abundance of ^{13}C-labeled glycolytic metabolites of cells treated with ^{13}C-glucose. n = 3.

(C) Abundance of ^{13}C-labeled intermediate metabolites in TCA cycle of cells treated with ^{13}C-glucose. n = 3.

Data are expressed as mean ± s.e.m; *$P < 0.05$, **$P < 0.01$, ***$P < 0.001$ by two-tailed Student's *t* test. ns, not significant.

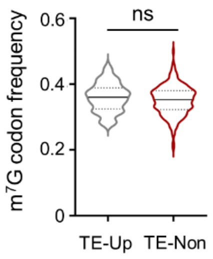

Figure S14 Codon frequency analysis of m^{7}G-tRNA decoded codons

Frequency analysis of m^{7}G-tRNA decoded codons in genes with increased translation (TE-up) and without translational change (TE-none) in *Prrx1*^{Cre};*Mettl1*$^{fl/fl}$ limb mesenchymal progenitors. *P* value was calculated by Mann–Whitney U test. ns, not significant.

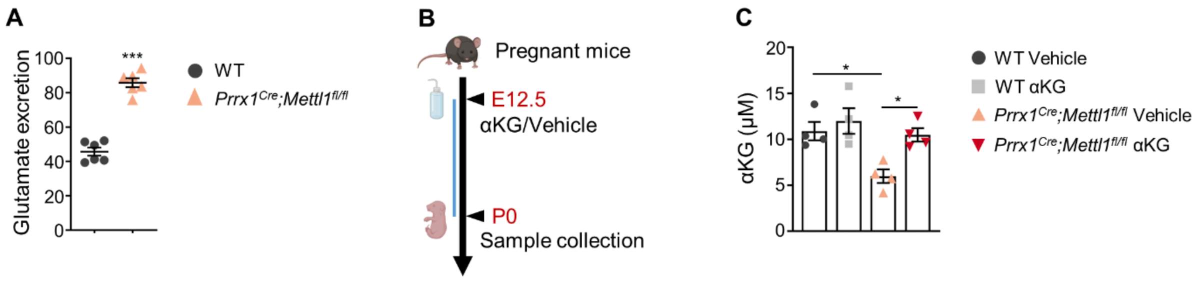

Figure S15 Supplementation of αKG to pregnant mice

(A) Measure of glutamate level in culture medium of WT and *Prrx1*^{Cre};*Mettl1*$^{fl/fl}$ limb mesenchymal progenitors. n = 6.

(B) Schematic representation of αKG supplementation.

(C) Measure of αKG level of cartilage tissue isolated from WT and *Prrx1*^{Cre};*Mettl1*$^{fl/fl}$ newborn mouse, supplemented with vehicle or αKG.

Data are expressed as mean ± s.e.m; *$P < 0.05$, **$P < 0.01$, ***$P < 0.001$ by two-tailed Student's *t* test (A) or one-way ANOVA with Tukey's post hoc test (C).

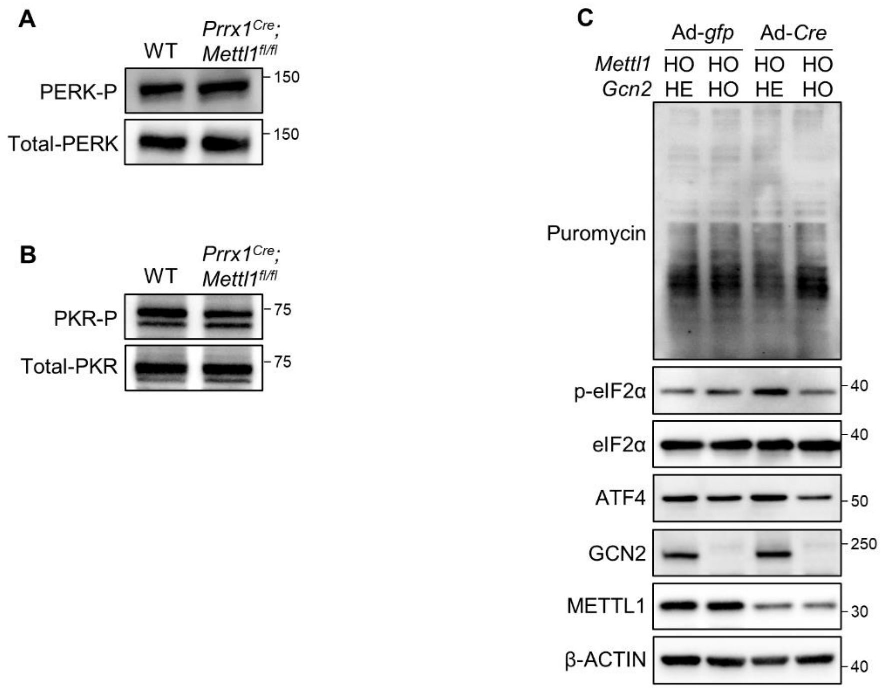

Figure S16 *Mettl1* removal activates ISR via GCN2

(A) Western blot analysis of phospho-PERK and PERK levels in WT and *Prrx1*^{Cre}*;Mettl1*$^{fl/fl}$ limb mesenchymal progenitors.

(B) Western blot analysis of phospho-PKR and PKR levels in WT and *Prrx1*^{Cre}*;Mettl1*$^{fl/fl}$ limb mesenchymal progenitors.

(C) Western blot analysis of ISR signaling and puromycin incorporation into nascent peptides in bone marrow SSCs isolated from *Mettl1*$^{fl/fl}$*;Gcn2*$^{+/-}$ and *Mettl1*$^{fl/fl}$*;Gcn2*$^{-/-}$ mice at 4 weeks of age, and infected with Ad-*gfp* or Ad-*Cre*. HE, heterozygous; HO, homozygous.

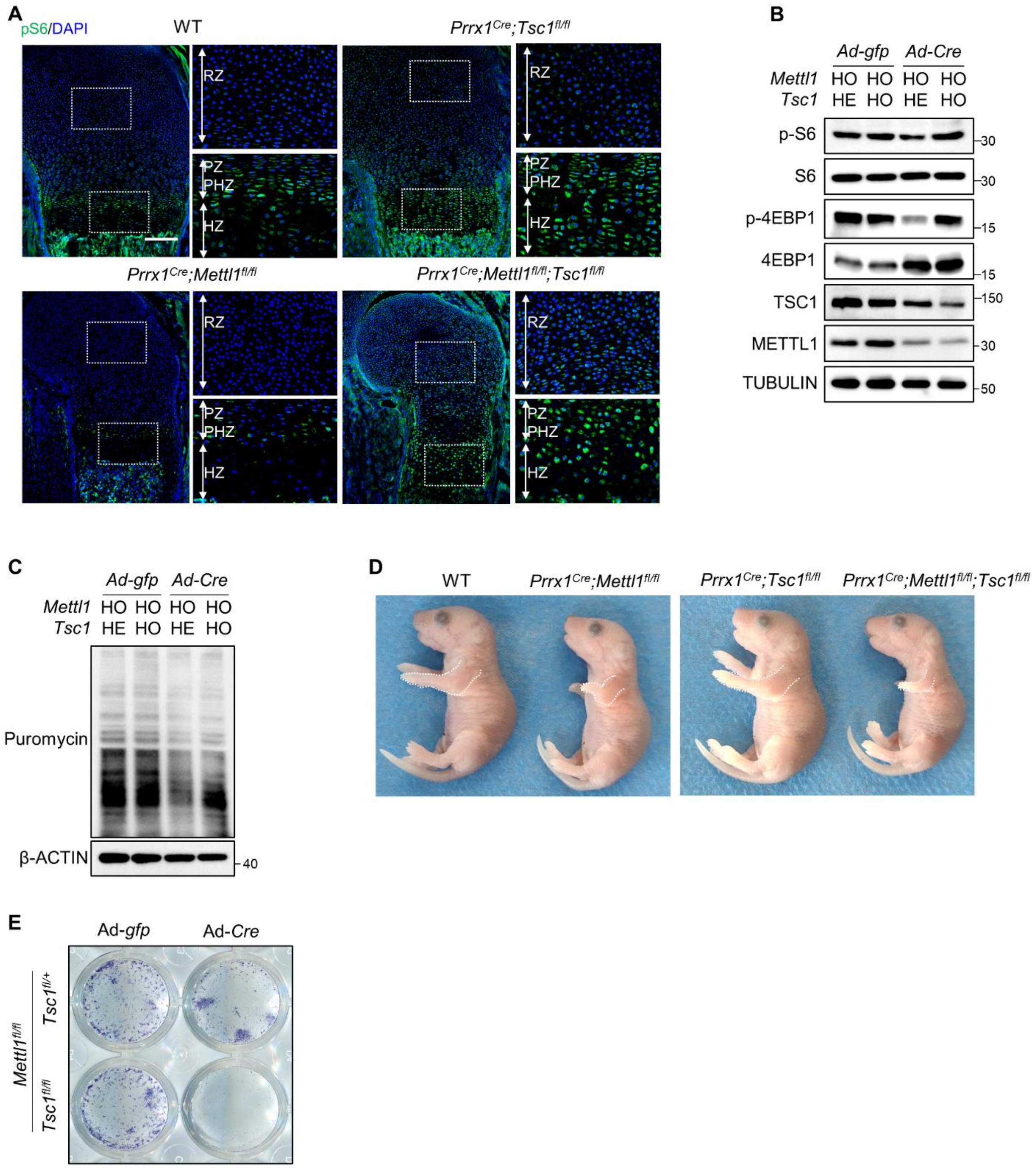

Figure S17 Promoting translation by mTORC1 activation aggravates bone defect of *Mettl1*-knockout mice

(A) Immunostaining of phospho-S6 in WT, *Prrx1*^{Cre};*Tsc1*$^{fl/fl}$, *Prrx1*^{Cre};*Mettl1*$^{fl/fl}$ and *Prrx1*^{Cre};*Mettl1*$^{fl/fl}$;*Tsc1*$^{fl/fl}$ mouse humerus at P0. Boxed areas were magnified. RZ, resting zone; PZ, proliferating zone; PHZ, pre-hypertrophic zone; HZ, hypertrophic zone. Scale bar, 200 μm.

(B) Western blot analysis of S6, p-S6, 4EBP1, p-4EBP1, TSC and METTL1 in bone marrow SSCs isolated from *Mettl1*$^{fl/fl}$;*Tsc1*$^{fl/+}$ and *Mettl1*$^{fl/fl}$;*Tsc1*$^{fl/fl}$ mice at 4 weeks of age, and infected with Ad-*gfp* or Ad-*Cre*. HE, heterozygous; HO, homozygous.

(C) Western blot analysis of puromycin incorporation into nascent peptides in bone marrow SSCs isolated from *Mettl1*$^{fl/fl}$*;Tsc1*$^{fl/+}$ and *Mettl1*$^{fl/fl}$*;Tsc1*$^{fl/fl}$ mice at 4 weeks of age, and infected with Ad-*gfp* or Ad-*Cre*. HE, heterozygous; HO, homozygous.
(D) Representative images of WT, *Prrx1*^{Cre}*;Tsc1*$^{fl/fl}$, *Prrx1*^{Cre}*;Mettl1*$^{fl/fl}$ and *Prrx1*^{Cre}*;Mettl1*$^{fl/fl}$*;Tsc1*$^{fl/fl}$ mice at P0.
(E) Representative images of ALP staining of bone marrow SSCs isolated from *Mettl1*$^{fl/fl}$*;Tsc1*$^{fl/+}$ and *Mettl1*$^{fl/fl}$*;Tsc1*$^{fl/fl}$ mice at 4 weeks of age, and infected with Ad-*gfp* or Ad-*Cre*.

Supplementary Table 1

Genes	Primers	Sequence (5'-3')	Product size
Prrx1^{Cre}/*Sp7*^{Cre} WT	Forward	ACTGGGATCTTCGAACTCTTTGGAC	WT = 397 bp
	Reverse	GATGTTGGGGCACTGCTCATTCACC	
Prrx1^{Cre}/*Sp7*^{Cre} Mut	Forward	CCATCTGCCACCAGCCAG	Mut = ~300 bp
	Reverse	TCGCCATCTTCCAGCAGG	
Mettl1-flox	Forward	ATACACACTCCTGGCCACTCCTTGT	WT = 379 bp<br>Mut = 465 bp
	Reverse	CCCTGGCTTCTAGGGTGTGAATCAG	
Wdr4 p.mut	Forward	GGGAGCGGTGCAGCAGGTAT	WT = 585 bp<br>Mut = 585 bp
	Reverse	GCAGCCCACAGCCCTGGATT	
Gcn2 WT	Forward	AAGCACTCAAGAAGCTGACATCACC	WT = 321 bp
	Reverse	ATCTATGCACGCTCTCTCAAGAAGC	
Gcn2 Mut	Forward	GATCATCTGCCCTCTAGTCTGTATG	Mut = 273 bp
	Reverse	TGTAGCCTGTCATCTCCATTCCTAG	
Tsc1-flox	Forward	GTCACGACCGTAGGAGAAGC	WT = 193 bp<br>Mut = 230 bp
	Reverse	GAATCAACCCCACAGAGCAT	

Supplementary Table 2

Genes	Sequence
tRNA-Val-AAC	TGTTTCCGCCCGGTTTCGAA
tRNA-Lys-CTT	AACGTGGGGCTCGAACCCAC
U6 snoRNA	TGGAACGCTTCACGAATTTG

Supplementary Table 3

Genes	Primers	Sequence (5’-3’)
Gapdh	Forward	TCATTGACCTCAACTACATG
	Reverse	TCGCTCCTGGAAGATGGTGAT
Runx2	Forward	GGTACTTCGTCAGCATCCTATCAG
	Common	GCTTCCGTCAGCGTCAACAC
Sp7	Forward	ATGGCGTCCTCTCTGCTTG
	Reverse	TGAAAGGTCAGCGTATGGCTT
Bglap	Forward	TTGGTGCACACCTAGCAGAC
	Reverse	ACCTTATTGCCCTCCTGCTT
Alp	Forward	AACCCAGACACAAGCATTCC
	Reverse	GCCTTTGAGGTTTTTGGTCA
Col1a1	Forward	TAGGCCATTGTGTATGCAGC
	Reverse	ACATGTTCAGCTTTGTGGACC
Col2a1	Forward	AGAACATCACCTACCACTGTAAGAACA
	Reverse	TGACGGTCTTGCCCCACTT
Col10a1	Forward	CCTTTCTGCTGCTAATGTTCTTGA
	Reverse	ATGCCTTGTTCTCCTCTTACTGGA
Mmp13	Forward	ACTTCTACCCATTTGATGGACCTT
	Reverse	AAGCTCATGGGCAGCAACA

Supplemental Methods

Antibodies	Source	Catalog
METTL1	Proteintech	Cat #:14994-1-AP; RRID: AB_2142013
Col10	Abcam	Cat #: ab58632; RRID: AB_879742
MMP13	Abcam	Cat #: ab39012; RRID:AB_776416
Sp7	Abcam	Cat #: ab209484; RRID:AB_2892207
TUBULIN	Proteintech	Cat #: 11224-1-AP; RRID:AB_2210206
WDR4	Proteintech	Cat #: 14766-1-AP; RRID:AB_2215387
Puromycin	Millipore	Cat #: MABE343; RRID:AB_2566826
7-methylguanosine (m7G)	MBL International	Cat #: RN017M; RRID:AB_2725740
Phospho-eIF2α (Ser51)	CST	Cat #: 3398; RRID:AB_2096481
eIF2α	CST	Cat #: 5324; RRID:AB_10692650
ATF4	Huabio	Cat #: ET1612-37
Phospho-GCN2	Abcam	Cat #: 75836; RRID:AB_1310260
GCN2	CST	Cat #: 3302S; RRID: AB_2277617
Phospho-PERK	Immunoway	Cat #: YP1005
PERK	CST	Cat #: 3192S; RRID:AB_2095847
Phospho-PKR	Abcam	Cat #: 32036; RRID:AB_777310
PKR	Proteintech	Cat #: 18244-1-AP; RRID:AB_2246451
β-ACTIN	Proteintech	Cat #: 20536-1-AP; RRID:AB_10700003
Phospho-S6 Ribosomal Protein (Ser235/236) (D57.2.2E)	CST	Cat #: 4858; RRID:AB_916156
Ribosome Protein S6	Santa cruz	Cat #: sc-74459; RRID:AB_1129205
Phospho-4E-BP1 (Thr37/46) (236B4)	CST	Cat #: 2855; RRID:AB_56083
4EBP1	Huabio	Cat #: ER62532

TSC1	Proteintech	Cat #: 29906-1-AP; RRID:AB_2918352
phospho-AMPKα (Thr172)	CST	Cat #: 2535; RRID:AB_331250
AMPKα	CST	Cat #: 2532; RRID:AB_330331
BCAT1	Proteintech	Cat #: 13640-1-AP; RRID:AB_2063569
NF2	Proteintech	Cat #: 21686-1-AP; RRID:AB_10792410
MYL9	Proteintech	Cat #: 15354-1-AP; RRID:AB_2147773
MYH9	Proteintech	Cat #: 11128-1-AP; RRID:AB_2147294
Alexa Fluor 488 Phalloidin	Thermo Fisher Scientific	Cat #: A12379; RRID:AB_2315147
Goat anti-Rabbit IgG secondary Ab HRP conjugated	Signalway	Cat #: L3012; RRID:AB_895483
Goat anti-mouse IgG secondary Ab HRP conjugated	Signalway	Cat #: L3032; RRID:AB_895481
Goat Anti-rabbit IgG (Cy3)	The Jackson ImmunoResearch	Cat #: 111-165-003; RRID:AB_2338000
Goat Anti-rabbit IgG (Alexa Fluor® 488)	The Jackson ImmunoResearch	Cat #: 111-545-003; RRID:AB_2338046
Goat Anti-rabbit IgG (Alexa Fluor® 647)	The Jackson ImmunoResearch	Cat #: 111-605-003; RRID:AB_2338072
**Bacterial and virus strains**		
Adeno-Cre	Genechem	N/A
Adeno-gfp	Genechem	N/A
**Chemicals, peptides and recombinant proteins**		
Puromycin	Sigma-Aldrich	Cat#P8833
Cycloheximide	Sigma-Aldrich	Cat#C7698
Alpha-ketoglutaric acid	Sigma-Aldrich	Cat#K1128

Alcian blue 8GX	Solarbio	Cat# A8140
Alizarin S	Sigma-Aldrich	Cat#A5533
EdU	Carbosynth	Cat#NE08701
Sulfo-Cyanine3 azide	Lumiprobe	Cat#D1330
Collagenase type II	Worthington-Biochem	Cat#LS004176
Dispase I	Sigma-Aldrich	Cat#D4693
Safranin O	Sigma-Aldrich	Cat#S2255
Fast Green	Sigma-Aldrich	Cat#F7252
Silver nitrate	Sigma-Aldrich	Cat#85193
Mitotracker Red CMXRos	Yeasen	Cat#40741ES50
MitoSOX	Invitrogen	Cat#M36008
TRIzol reagent	Invitrogen	Cat#15596026
NSC23766 trihydrochloride	MedChemExpress	Cat#HY-15723A
CN04 (Rho/Rac/Cdc42 Activator I)	Cytoskeleton	Cat#CN04-A
**Critical commercial assays**		
TUNEL assay kit	Promega	Cat#G3250
BCIP/NBT staining kit	Beyotime	Cat#C3206
ALP assay kit	Beyotime	Cat#P0321
PrimeScript RT reagent Kit with gDNA Eraser	Takara	Cat#RR047A
SYBR Premix Ex Taq II	Takara	Cat#1725121
Glucose assay kit	Nanjing jiancheng Bioengineering Institute	Cat#F006-1-1
aKG assay kit	Abcam	Cat#ab83431

Alcian blue staining kit	Solarbio	Cat#G1560
H&E staining kit	Solarbio	Cat#G1120
Seahorse XF cell mito stress test kit	Agilent	Cat#103015-100
Seahorse XF glycolytic rate assay kit	Agilent	Cat#103344-100
**Deposited data**		
TRAC-seq of limb progenitors from WT and *Prrx1*^{Cre};*Mettl1*$^{fl/fl}$ mice	This paper	https://ngdc.cncb.ac.cn/gsa/browse/CRA012901
Ribosome profiling results	This paper	https://ngdc.cncb.ac.cn/gsa/browse/CRA012883
Untargeted metabolomics	This paper	https://ngdc.cncb.ac.cn/omix: accession no. OMIX005049
**Experimental models: Organisms/strains**		
Mettl1$^{fl/+}$ mice	Biocytogen	N/A
Wdr4$^{R215L/+}$ mice	Biocytogen	N/A
Prrx1^{Cre} mice	The Jackson Laboratory	Stock No: 005584
Sp7^{Cre} mice	The Jackson Laboratory	Stock No: 035391
Gcn2$^{+/-}$ (*Eif2ak4*$^{+/-}$) mice	GemPharmatech	Stock No: T012642
Tsc1$^{fl/+}$ mice	The Jackson Laboratory	Stock No: 005680
**Oligonucleotides**		
See Table S1-S3 for a list of primers.		
**Software and algorithms**		
Fiji/ImageJ	ImageJ	https://imagej.nih.gov/ij/
FV10-ASW 3.1	Olympus	http://www.olympus.com.cn/
FlowJo 10	BD Biosciences	https://www.flowjo.com/

μCT50	SCANCO Medical	N/A
ChemiDoc	Biorad	https://www.bio-rad.com/en-jp/category/chemidoc-imaging-systems?ID=NINJ0Z15
GraphPad Prism	Prism	https://www.graphpad-prism.cn/prism.html
SPSS 20.0	IBM	https://www.ibm.com/spss
Imaris 8.1	Nikon	https://imaris.oxinst.com/
OsteoMeasure	OsteoMetrics	https://www.osteometrics.com/
Seahorse Wave	Agilent	https://www.agilent.com/en/product/cell-analysis/real-time-cell-metabolic-analysis/xf-software/seahorse-wave-desktop-software-740897
